# Selection and Validation of Reference Genes for Gene Expression Analysis in Switchgrass (*Panicum virgatum*) Using Quantitative Real-Time RT-PCR

**DOI:** 10.1371/journal.pone.0091474

**Published:** 2014-03-12

**Authors:** Jacinta Gimeno, Nicholas Eattock, Allen Van Deynze, Eduardo Blumwald

**Affiliations:** Department of Plant Sciences, University of California Davis, Davis, California, United States of America; Nazarbayev University, Kazakhstan

## Abstract

Switchgrass (*Panicum virgatum*) has received a lot of attention as a forage and bioenergy crop during the past few years. Gene expression studies are in progress to improve new traits and develop new cultivars. Quantitative real time PCR (qRT-PCR) has emerged as an important technique to study gene expression analysis. For accurate and reliable results, normalization of data with reference genes is essential. In this work, we evaluate the stability of expression of genes to use as reference for qRT-PCR in the grass *P. virgatum*. Eleven candidate reference genes, including *eEF-1α*, *UBQ6*, *ACT12*, *TUB6*, *eIF-4a*, *GAPDH*, *SAMDC*, *TUA6*, *CYP5*, *U2AF*, and *FTSH4*, were validated for qRT-PCR normalization in different plant tissues and under different stress conditions. The expression stability of these genes was verified by the use of two distinct algorithms, geNorm and NormFinder. Differences were observed after comparison of the ranking of the candidate reference genes identified by both programs but *eEF-1α*, *eIF-4a*, *CYP5* and *U2AF* are ranked as the most stable genes in the samples sets under study. Both programs discard the use of *SAMDC* and *TUA6* for normalization. Validation of the reference genes proposed by geNorm and NormFinder were performed by normalization of transcript abundance of a group of target genes in different samples. Results show similar expression patterns when the best reference genes selected by both programs were used but differences were detected in the transcript abundance of the target genes. Based on the above research, we recommend the use of different statistical algorithms to identify the best reference genes for expression data normalization. The best genes selected in this study will help to improve the quality of gene expression data in a wide variety of samples in switchgrass.

## Introduction

The instability of petroleum sources and threat of global climate change have incited a push towards locally produced renewable energy sources. Recent scientific developments have allowed the conversion of renewable biologic materials to liquid or gaseous fuels. Of particular interest is the saccharification of cellulose cell wall, the principal component of plants, to its sugar components that can be fermented into high value liquid ethanol with the potential to displace a substantial portion of petroleum based fuels.

Switchgrass (*Panicum virgatum L.*) is a perennial warm-season grass native to North America and a productive C4 species. Used initially as a forage crop, switchgrass is currently being pursued as a dedicated cellulosic biofuel feedstock in the Midwestern and Southeastern United Sates. Switchgrass has been shown to produce 540% more renewable energy than energy consumed in its production and has significant environmental benefits related to soil conservation and low greenhouse gas emission [1]. Its perennial nature allows efficient nutrient cycling to below ground organs for the subsequent seasons' growth, a characteristic that allows substantial annual harvests with low nutrient and water requirements [2].

As a result of the increasing interest in this species, numerous studies are under way in switchgrass to understand the basis of water and nutrient- use efficiency [3]–[6] and the mechanisms of cell wall synthesis [7]–[8]. Moreover, an increasing number of genomic resources have been developed including SSR markers [9]–[11], miRNAs [12], ESTs, microarrays [13]–[16] and a transformation system [17].

To verify the function of a candidate gene, the analysis of mRNA expression patterns is one of the most used molecular techniques. Real-time RT-PCR (also referred to as quantitative RT-PCR or qRT-PCR) has become a popular approach for the analysis of gene expression, its main advantages being a high sensitivity, specificity and broad quantification range [18]. The accuracy of qRT-PCR is strongly influenced by the stability of the internal reference genes used for data normalization [19], [20]. It requires the use of constitutively expressed genes as internal controls to correct for variability associated with the different steps of the experimental procedure. The expression levels of these genes should remain constant between different tissues and/or in response to different environmental stimuli. Though there are several genes frequently used as HKGs (Housekeeping genes) (*18S sRNA, actin, tubulin*), many studies demonstrate that the expression of these genes is not always stable and the selection of alternative genes with stable expression profiles must be identified in each organism across experimental conditions prior to the use of real-time PCR [21]. Several statistical approaches have been proposed for evaluating reference genes such geNorm [22], NormFinder [23] and Best-Keeper [24]. These approaches are based on different statistical algorithms and employ multiple reference genes as the best strategy for normalization of qPCR results.

Until now, the number of studies on gene expression in switchgrass is scant and few genes are used as reference genes for normalization [7], [25]–[28]. A recent switchgrass transcriptome analysis [16] identified a set of stably expressed genes by measuring the transcript levels in all major organ systems at different stages of development. This group of genes could be a source of candidate reference genes but will need to be evaluated as reference genes for each experimental condition.

Given the lack of information on appropriate reference genes in switchgrass, eleven HKGs, commonly used in plant gene expression studies, were selected in order to assess them as internal controls. These genes are elongation factor 1α (*eEF-1α*), ubiquitin (*UBQ6*), actin (*ACT12*), β-tubulin (*TUB6*), eukaryotic initiation factor (*eIF-4a*), glyceraldehyde-3-phosphate dehydrogenase (*GAPDH*), s-adenosyl methionine decarboxylase (*SAMDC*), α-tubulin (*TUA6*), cyclophilin (*CYP5*), splicing factor (*U2AF*) and FtsH protease 4 (*FTSH4*). The stability of expression of these candidate reference genes was tested in different switchgrass tissues and under different stress conditions using both geNorm [22] and NormFinder [23] algorithms.

In addition, we provide a detailed expression analysis of seven putative homologues of the cellulose synthase gene family in switchgrass (SGCesAs), as an example for the genes involved in cellulose biosynthesis. Defining the pattern of their expression in different tissues and under different stress conditions may lead to their utilization towards improving cellulose production in energy crops, main sources of cellulosic ethanol. To illustrate the usefulness of the most stable reference genes identified in this work, the best combinations of reference genes were used to perform the expression normalization. Therefore, this study provides useful guidelines and starting point for reference gene selection for expression analysis using qRT-PCR techniques in *P.virgatum*.

## Materials and Methods

### Plant material

Switchgrass (*Panicum virgatum* L. cv. Alamo) seeds on water were cold-treated at 4°C overnight to synchronize germination and planted in the greenhouse in flats filled with UC mix (50% washed sand, 50% sphagnum peat moss). After germination, seedlings of 35–40 cm where transplanted to 7.6-liter pots filled with the UC mix.

Plants were grown in the greenhouse under 14 h photoperiod at 30°C day and 18°C night and the relative humidity was maintained between 40 and 70%. Irrigation was applied in early-morning under a regime of two days fertilization (N:P:K = 261∶30∶326 ppm) followed by one day of deionized water. Plants received 500 ml per day, the amount of water necessary to maintain the field capacity.

### Stress treatments and tissue collection

Samples were collected from different tissues and under different stress conditions. Tissue from leaves, stems, rachis and roots was collected from three-month old plants growing under well-watered conditions. Plants to be treated with different abiotic stresses were grown for four weeks under field capacity after transplanting. Plants were subjected to drought treatments by reducing the irrigation to 250 ml per day (50% water) for one week. Salt stress treatment was applied by a progressive increase of the salt concentration in the solution (40, 120, 200 and 300 mM NaCl). Four days of salt treatment were followed by one day of fertilization. Last salt solution concentration was applied for seven days. For cold and heat shock treatments plants were transferred to 4°C and 42°C respectively for 48 h. To apply wounding stress, the upper leaf of a tiller was mechanically wounded 3–5 times by a hemostat perpendicular to the mid-vein located two-thirds from the tip of the leaf. The leaf area wounded was approximate the 10% of the leaf surface. Tissue was collected after 24 hours. Flooding treatment was applied for 30 days by maintaining a water level above the soil surface. Tissue samples were collected from the last fully expanded leaf after each stress treatment. All 10 samples were collected from three replicate plants, giving a total of 30 samples comprised of 12 tissue-specific samples and 18 abiotic stress treatment samples. Samples were immediately frozen in liquid nitrogen and stored at −80°C until RNA extraction.

### Total RNA isolation and cDNA synthesis

A total of 30 mg of frozen plant material was ground in eppendorf tubes using 3.2 mm stainless steel beads and a shaker SO-10M (Fluid Management, Sassenheim, Netherlands). Total RNA was extracted using the RNeasy Mini kit (Qiagen, Valencia, USA) with the addition of an on-column DNase I digestion according to the manufacturer's instructions. RNA sample concentration and quality was determined using the NanoDrop ND-1000 spectrophotometer (NanoDrop Technologies, Wilmington, DE, USA). The quality of RNA samples was also assessed by 1% agarose gel electrophoresis.

First-strand cDNA was synthesized from 1 μg of total RNA in a total volume of 20 μl per reaction using the QuantiTect Reverse Transcription kit (Qiagen, Valencia, USA) following the manufacturer's protocol. RNA quantity and quality were verified using 1% agarose gel electrophoresis after the genomic DNA elimination step of this protocol. cDNAs were diluted to a final volume of 400 μl to be used on the quantitative real-time RT-PCR reactions (qRT-PCR).

### Selection of candidate reference genes, primer design and validation

A group of genes commonly used as reference genes in different plant species were selected to be evaluated as control genes in *P. virgatum*. Rice protein sequences from the potential reference genes were used as a query sequence for a TBLASTN search of the switchgrass GenBank EST database. Selected switchgrass ESTs were then used to query the non-redundant protein database using BLASTX for identity verification. The gene name, EST accession number and gene ontology are shown in Table 1. Specific primers for qRT-PCR were designed using the Primer Express 3.0 software (PE Applied Biosystems, Foster City, USA) using the default parameters for a real-time PCR assay. Amplicon lengths varied from 90 to 100 bp, melting temperatures were between 58–60°C and primer lengths between 18–24 bp (Table 2). The primers were screened for hairpins, dimer formation, and target specificity by BLASTN against the nr databank. A pool of cDNA from 30 samples was used to perform the qRT-PCR reactions to determine the primer efficiency. The entire raw fluorescence data was used to test each primer pair amplification with the Excel workbook entitled Data Analysis for Real-Time PCR (DART-PCRv.1.0) [29]. All primer amplification efficiencies were between 97 and 110% (Table 2). Agarose gel electrophoresis and melting curve of the amplification products of each candidate gene were analyzed in order to verify that a single PCR product was amplified for each set of primers.

**Table 1:** Description of candidate reference genes selected for evaluation of expression stability in *Panicum virgatum*.

Gene Name^a^	Gene description	GeneBank accession number db EST^b^	Arabidopsis ortholog locus	Rice TIRG identifier^c^	Rice TBLASTN Score	E-value ID (%)
***eEF-1α***	Eukariotic elongation factor 1-α	GR876801	At5g60390	LOC_Os03g08020	644/0.0	308/313 (98%)
***UBQ6***	Ubiquitin 6	FE609298	At2g47110	LOC_Os01g22490	246/1e-65	153/155 (98%)
***ACT12***	Actin 12	GR878265	At3g46520	LOC_Os3g50885	620/3e-177	296/313 (94%)
***TUB6***	Tubulin beta-6	GR880018	At5g12250	LOC_Os05g34170	607/3e-173	304/306 (99%)
***eIF-4a***	Eukaryotic initiation factor 4a	GR877213	At1g54270	LOC_Os02g05330	605/1e-172	293/304 (96%)
***GAPDH***	Glyceraldehyde-3-phosphate dehydrogenase C1	GR879471	At3g04120	LOC_Os08g03290	550/5e-158	281/303 (92%)
***SAMDC***	S-adenosyl methionine decarboxylase	FL72288	At3g02470	LOC_Os02g39790	492/7e-139	247/271 (91%)
***TUA6***	Tubulin alpha-6	GR879415	At4g14960	LOC_Os11g14220	626/4e-179	320/327 (97%)
***CYP5***	Cyclophilin 5	FE633090	At2g29960	LOC_Os06g49480	325/8e-89	179/225 (79%)
***U2AF***	Splicing factor U2af	FL907910	At5g42820	LOC_Os05g48960	384/2e-106	177/185 (95%)
***FTSH4***	Ftsh protease 4	FL791612	At2g26140	LOC_Os01g39260	451/3e-126	238/244 (37%)

**Note**: **a**. All Switchgrass sequences were named according on similarity to *Arabidopsis thaliana* proteins determined with BLASTX. **b**. Accession number of the most similar EST to the rice protein according to the Switchgrass GenBank dbEST. **c**. TIGR rice genome identifier of the rice proteins used to identify Switchgrass reference genes sequences via TBLASTN among the Switchgrass GenBank dbEST.

**Table 2:** Primer sequences and amplicons of the 11 candidate reference genes evaluated in this study.

Gene name	Gene description	Sequence of forward (F) and reverse (R) primers	Length of the amplified fragment (bp)	Primer efficiency
***eEF-1α***	Eukariotic elongation factor 1-α	F 5′-CGGTTGGTCGTGTGGAGACT-3′	100	1.028
		F 5′-TGGTGCATCTCAACAGACTTCAC-3′		
***UBQ6***	Ubiquitin 6	F 5′-AGAAGCGCAAGAAGAAGACG-3′	93	1.020
		F 5′-CCACCTTGTAGAACTGGAGCA-3′		
***ACT12***	Actin 12	F 5′-CAGCCATCCATGATCGGTATG-3′	100	0.990
		F 5′-TGCCGTACAGGTCCTTTCTGA-3′		
***TUB6***	Tubulin beta-6	F 5′-GAGGAGTACCCTGATCGGATGA-3′	90	1.017
		F 5′-GGTTGCATTGTATGGCTCAACA-3′		
***eIF-4a***	Eukaryotic initiation factor 4a	F 5′-TGATGTCATTCAGCAAGCACAA-3′	95	0.967
		F 5′-GGCATTCAACCAGGCCATAG-3′		
***GAPDH***	Glyceraldehyde-3-phosphate dehydrogenase C1	F 5′-TCCTGAATTGAATGGCAAGCT-3′	100	0.993
		R 5′-GGAGGCCCCCTTCTCGAT-3′		
***SAMDC***	S-adenosyl methionine decarboxylase	F 5′-AAGACCTGCGGGACTACCAA-3′	100	1.079
		R 5′-CACGTGAGTATTTAACAGCAGCAA-3′		
***TUA6***	Tubulin alpha-6	F 5′-ACTCCCTCCTTGAGCACACTGA-3′	100	1.047
		F 5′-GTGTAGGTTGGGCGCTCAAT-3		
***CYP5***	Cyclophilin 5	F 5′-CACTACAAGGGAAGCACATTCCA-3′	90	1.039
		F 5′-TTCACCACCCCTTCCATCAC-3′		
***U2AF***	Splicing factor U2af	F 5′-GGGTCAACTGCCCTTTTTACTTC-3′	100	0.968
		F 5′-AGCACAAGAGTCGGCGATATG-3′		
***FTSH4***	Ftsh protease 4	F 5′-TGGATGGCTTTAAGCAGAATGA-3′	95	1.046
		F 5′-CAAAACGCCCAGGTCTGACT-3′		

### Development of specific primers for *CesA* genes in switchgrass

The amino acid sequences of *Oryza sativa* cellulose synthase gene family were used as query sequences for TBLASTN search of the switchgrass GenBank EST database. TIGR identifiers of the rice genes used are: Os01g54620, Os03g59340, Os07g10770, Os05g08370, Os07g24190, Os03g62090, Os07g14850, Os09g25490, Os10g32980 and Os06g39970. EST sequences obtained (>80% identity) were assembled by CAP3 assembly tool [30] with default parameters. The resulting partial DNA consensus sequences after assembly (File S1) were used to design specific primers on the HVR-II region to discriminate the different members of the family and analyze the expression patterns. Primer design and evaluation (Table S1) and qRT-PCR were performed as described previously.

### Real-time RT-PCR analysis

The real-time RT-PCRs were performed using the StepOnePlus Real-Time PCR System (Applied Biosystems, Foster City, USA). Each PCR reaction contained 7.5 μl of 2× Fast SYBR Green master mix (Applied Biosystems, Foster City, USA), 2 μl of the diluted cDNA reaction mixture (corresponding to 5 ng of starting amount of RNA) and 200 nM of each primer in a total reaction volume of 15 μl. PCR reactions were performed under the following conditions: 10 min at 95°C and 40 cycles of the one step thermal cycling of 3 s at 95 °C and 30 s at 60°C in a 96-well reaction plate. Specificity of PCR reactions was verified by melting curve analysis of each sample amplified product. Each real-time PCR reaction was performed in triplicate (technical replicates) on three individual plants (biological replicates).

### Analysis of reference gene expression stability

The fluorescence raw data, generated by the Applied Biosystems StepOne software 2.0 (PE Applied Biosystems, Foster City, USA), were exported to the DART-PCR v.1.0 [29]. This programmed Excel workbook enables the rapid calculation of threshold cycles, amplification efficiencies and *R_0_* (raw expression values) for every sample. Differences in amplification efficiency were assessed using one-way analysis of variance (ANOVA), based upon the null hypotheses: (i) that amplification efficiency is comparable within sample groups (outlier detection) and (ii) that amplification efficiency is comparable between sample groups (amplification equivalence). Outliers identified were omitted prior to further analysis.

Relative quantities were calculated in Microsoft Excel using the highest expression value as calibrator, and then they were imported to the gene expression stability program geNorm 3.5 [22], [31]. This program estimates an expression stability value (M) for each gene as the average pairwise variation for a particular gene with all the other tested reference genes included in the analysis. The M-value measure relies on the principle that the expression ratio of two ideal internal control genes is identical in all samples, regardless of the experimental conditions or treatments. Genes with the lowest expression stability are removed and a new M value for each of the remaining genes is calculated until only two genes remain. The geNorm program also establishes the minimal number of reference genes required for calculating an accurate normalization factor (NF). The NF_n_ is calculated as the geometric mean of the expression level of the *n* most stable genes and recalculated adding the next most stable gene. Finally, geNorm calculates the pairwise variation, V_n_/V_n+1_, between two sequential normalization factors, NF_n_/NF_n+1_, reflecting the effect of the addition of a new gene on the normalization factor. A pair-wise variation (V) value higher than 0.15 implies that the added gene has a significant effect on normalization and should be included for calculation of a reliable normalization factor. Additional reference genes should be included to the normalization factor until the added gene has no significant effect. The optimum number of genes is the lowest number of genes with an acceptably low V_n_/V_n+1_.

NormFinder software [23], [32] was also used to evaluate the candidate normalization genes. The expression values obtained from DART-PCR were used as inputs for calculations. NormFinder has the ability to measure the gene expression stability taking into account intra- and inter-variations in defined samples groups or treatments. The combination of these estimates provides a direct measure of the variation in expression for each gene. This function determinates the best combination of two reference genes for normalization. The use of the two reference genes in combination will be, in most cases, more accurate than just using the most stable gene. The most stable candidate genes within and between the groups are those with the lowest variation values.

### Determination of *SGCesA* expresión profile

The expression profile of the putative SGCesA was calculated in different tissue (leaf, stem, rachis and root) and leaves of plants under different abiotic stress conditions. Normalization factors were calculated as the geometric mean of the expression values (R_0_) of the reference genes tested. Relative expression levels of the target genes are calculated by dividing the expression value of the target gene by the normalization factor. The measures of expression were made in three biological replicates of three technical repeats per sample.

## Results

### Selection of candidate reference genes in *P. virgatum*, primer design and amplification specificity

**Figure 1:**
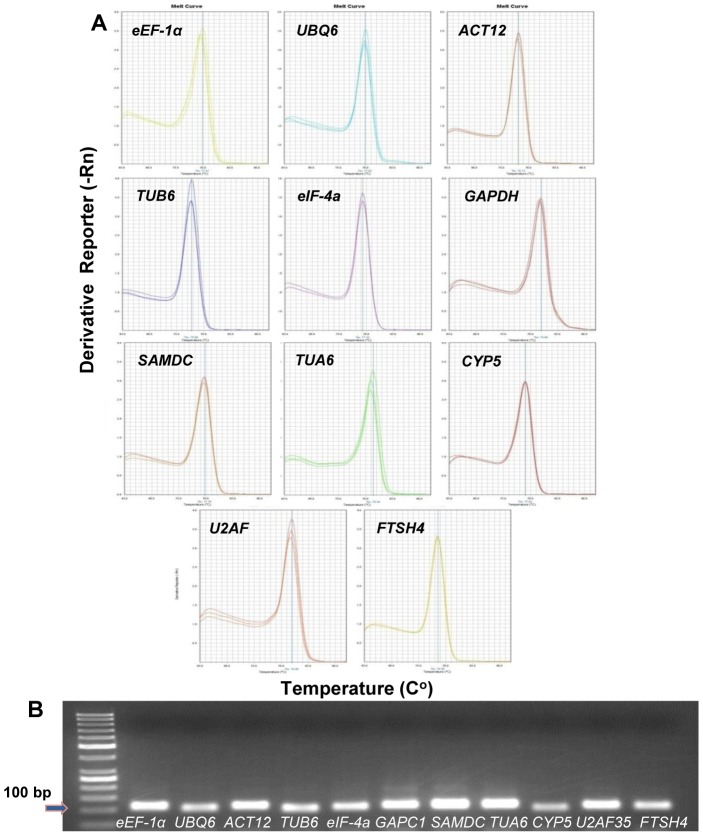
Specificity of real-time RT-PCR amplification. **A**) Melting curve of the 11 reference genes showing a single pick (each including three technical replicates of the cDNA pool of the total samples used in this study). **B**) Agarose gel (1.5%) showing amplification of a specific PCR product of the expected size for each gene tested in this study.

Based on searches of the literature a group of previously used control genes in plants was selected [33]–[36]. The sequences for 15 candidate reference genes were identified after performing a TBLATN among the switchgrass GenBank ESTdb. From three to four primer pairs per gene were designed for qRT-PCR and finally, primer pairs for 11 genes were selected on the basis of their amplification efficiency and single PCR product. The candidate reference genes encode eEF-1α (elongation factor 1α), UBQ6 (ubiquitin 6), ACT12 (Actin 12), TUB6 (β-tubulin 6), eIF-4a (eukaryotic initiation factor 4a), GAPDH (glyceraldehyde-3-phosphate dehydrogenase C1), SAMDC (s-adenosyl methionine decarboxylase), TUA6 (α-tubulin 6), CYP5 (cyclophilin 5), U2AF (Splicing factor U2af) and FTSH4 (FtsH protease 4) are described in Table 1. The products of these genes are associated with a wide variety of biological functions. Specificity of real-time PCR products was confirmed by the presence of a single peak in the melting curve and the presence of a single band with the expected size (90 to 100 bp) after agarose gel electrophoresis and GelRed nucleic acid staining (Figure 1). The primer sequences and amplification efficiencies are indicated in Table 2.

The transcript abundance of these 11 potential reference genes was analyzed by qRT-PCR across a series of three biological repeats of 10 different samples obtained from: leaves from plants under different abiotic stress conditions and leaves, stems, roots and rachis from well watered plants.

### Expression profile of the reference genes

**Figure 2:**
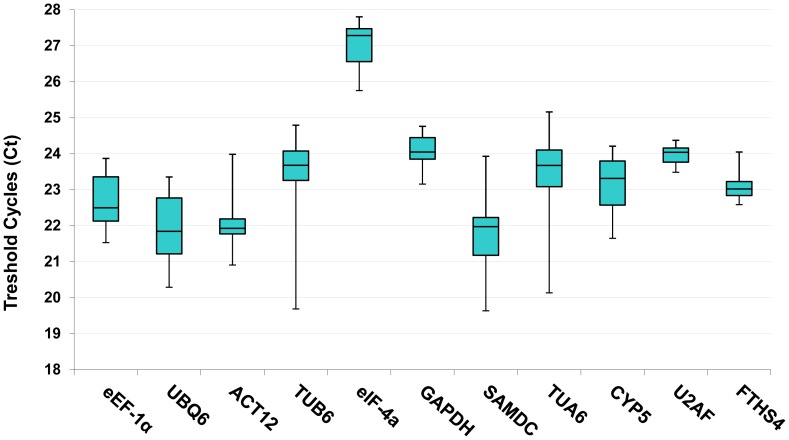
Cycle threshold (Ct) values of the candidate reference genes across the experimental samples. Box-plot graph of Ct value shows the median values as lines across the box. Lower and upper boxes indicating the 25^th^ percentile to the 75^th^ percentile. Whiskers represent the maximum and minimum values.

The cycle threshold (Ct) values in the qRT-PCR reactions are defined as the cycle at which the fluorescent signal is significantly different from the background and is used to identify the differences in transcript expression levels. The Ct mean values for the 11 reference genes on the 10 samples under study were used to compare the expression rates between genes and within the set of samples. The data analysis showed that candidate reference genes were moderately abundant and exhibited a wide range of expression levels in all tested samples. As can be seen in Figure 2, most of the selected genes presented Ct values that range from 19.6 to 27.1, while majority of the values were distributed between 21 and 24. The expression variation of an individual gene within all samples was smaller in *U2AF, FTSH4* and *GAPDH* and higher in *TUB6, TUA6* and *SAMDC*. The lowest and highest variation values were 0.88 and 5.10 cycles corresponding to *U2AF* and *TUB6*, respectively. The most highly expressed genes were *UBQ6* and *SAMDC* having a mean Ct value of 21 cycles. *eIF-4a* showed the lowest transcript abundance with a Ct of 27 cycles. Although some of these genes showed a low expression variation between the tested samples, it would be necessary to perform further analyses in order to identify the most suitable combination of these genes candidates for normalizing gene expression under certain experimental conditions.

### Expression stability analysis

Two publicly available software tools, geNorm [22] and NormFinder [23] were used to analyze the gene expression stability and the number of reference genes necessary for accurate gene-expression profiling. Expression values were obtained using the DART-PCR analysis excel software which takes in account the amplification efficiency of each gene to obtain relative quantities [29].

**Figure 3:**
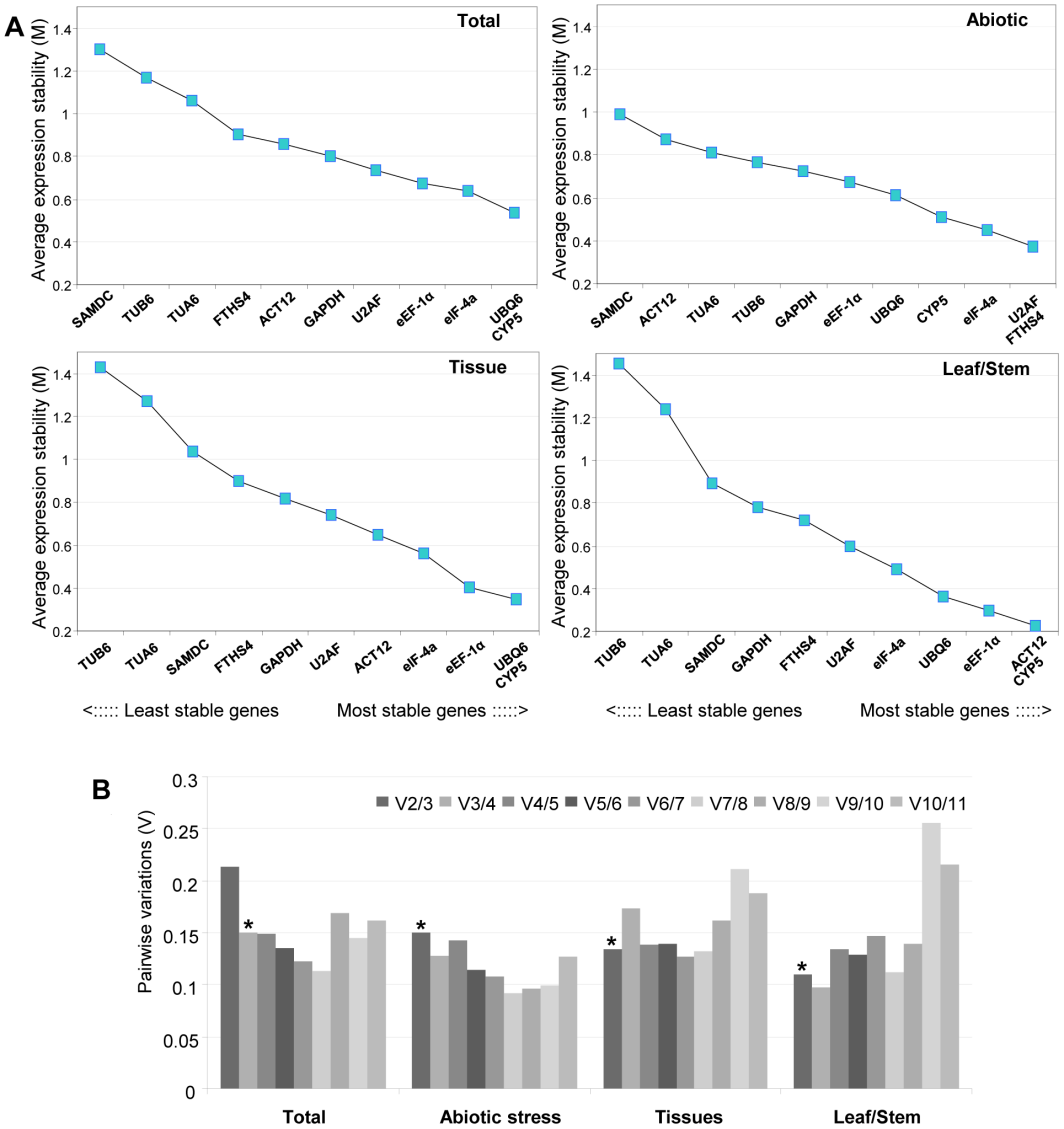
Gene expression stability values (M) and pairwise variation (V) of the candidate reference genes calculated by geNorm. A. Ranking of the gene expression stability performed in all the samples, abiotic stress samples, tissue samples and leaf and stem samples. The least stable genes are on the left and the most stable genes on the right. B. Pairwise variation (V_n_/V_n+1_) was analyzed between the normalization factors NFn and NFn+1. Asterisk indicates the optimal number of reference genes required for normalization.

We used geNorm to analyze gene expression stability across different sets of samples: 1) all the samples, 2) abiotic stress samples 3) total tissues samples and 4) leaves and stems tissue samples. The genes were ranked accordingly to the average expression M values (Figure 3A). The 11 genes used for the analysis showed high expression stability and presented an M value lower than the cutoff established by Vandesompele et al. [22] (M<1.5), otherwise the most stable reference genes were not identical in all the sets of samples under study. When all the samples were taken together, the average expression stability value (M) for *UBQ6* and *CYP5* was the lowest, and that of the *SAMDC* was the highest, indicating that *UBQ6* and *CYP5* had the most stable expression and that *SAMDC* was the most variably expressed of the 11 candidate genes. The genes *U2AF* and *FTSH4* had the highest expression stability in samples under abiotic stress conditions and once again, *SAMDC* was the least stable gene. When only plant tissue samples were considered, *UBQ6* and *CYP5* were the best candidates for normalization and in the subset of samples composed by samples from leaves and stems the most stable genes were *ACT12* and *CYP5* while in both cases, *TUB6* was the worst gene (Figure 3A).

The geNorm program was also used to calculate the optimal number of reference genes required for accurate normalization. Pair-wise variation analysis (Figure 3B) showed that the ideal number of reference genes may be different for distinct set of samples. When evaluating pairwise variation in all plant samples data set, V2/3 value was 0.213 and V3/4 was 0.15. The V3/4 value is equal to the cut-off value of 0.15, indicating that the use of the three most stable reference genes, *UBQ6, CYP5* and *eIF-4a*, is sufficient for accurate normalization. On the other hand, only two control genes are sufficient for accurate normalization in tissues (*UBQ6* and *CYP5*) or tissues under abiotic stress (*U2AF* and *FTHS4*), as indicated by the V2/3 values lower or equal than 0.15 in Figure 3B.

Stability of expression was then re-analyzed using the NormFinder algorithm that ranks the best candidate reference genes according to their minimal combined inter- and intra-group variation of expression for normalization factor (NF) calculation. Results are summarized in Table 3. When all the 30 samples data set or tissue samples subset were considered, *eIF-4a* and *eEF-1α* were identified as the most stable genes with a stability value between 0.379 to 0.299. The best combination of two genes was *eEF-1α* and *ACT12* which improved the stability value to 0.224 and 0.206, respectively. Although *eEF-1α* was again identified as the most stable gene for the subset of samples from leaves and stems, a different best combination of two genes was recommended for this set, for which NormFinder proposed *eEF-1α* and *CYP5*. Finally, in the samples including vegetative tissue under abiotic stress, NormFinder identified *FTHS4* as the most stably expressed gene and the best combination of two genes recommended was *CYP5* and *FTHS4*. Out of the four datasets, *SAMDC* and *TUB6* were the most often identified as the least stable reference genes.

**Table 3:** Expression stability values of *P. virgatum* candidate reference genes as calculated by the NormFinder software.

Total		Abiotic		Tissue		Leaf/Stem	
Ranking	Stability value	Ranking	Stability value	Ranking	Stability value	Ranking	Stability value
*eIF-4a*	0.348	*FTHS4*	0.216	*eEF-1α*	0.299	*eEF-1α*	0.200
*eEF-1α*	0.379	*eIF-4a*	0.324	*eIF-4a*	0.379	*CYP5*	0.222
*CYP5*	0.385	*U2AF*	0.328	*ACT12*	0.398	*eIF-4a*	0.314
*U2AF*	0.396	*CYP5*	0.339	*U2AF*	0.427	*ACT12*	0.362
*GAPDH*	0.498	*GAPDH*	0.365	*CYP5*	0.447	*UBQ6*	0.427
*ACT12*	0.500	*eEF-1α*	0.427	*UBQ6*	0.552	*U2AF*	0.498
*FTHS4*	0.548	*TUB6*	0.476	*GAPDH*	0.693	*FTHS4*	0.795
*UBQ6*	0.578	*TUA6*	0.556	*FTHS4*	0.822	*GAPDH*	0.838
*TUA6*	0.842	*ACT12*	0.568	*SAMDC*	1.087	*SAMDC*	1.026
*TUB6*	0.929	*UBQ6*	0.582	*TUA6*	1.168	*TUA6*	1.390
*SAMDC*	1.025	*SAMDC*	0.682	*TUB6*	1.351	*TUB6*	1.492
**Best combination**	**Stability value**	**Best combination**	**Stability value**	**Best combination**	**Stability value**	**Best combination**	**Stability value**
*eEF-1^α^ ACT12*	0.224	*CYP5 FTHS4*	0.166	*eEF-1^α^ ACT12*	0.206	*eEF-1α CYP5*	0.174

**Note**: Genes are ranked according to their stability values from the most stable genes to the least stable.

### Reference genes validation by quantification of *SGCesAs* expression profile with different normalization factors

In order to validate the use of the genes selected by geNorm and NormFinder algorithms as reference genes, we examine the effect of using different HKGs on the qRT-PCR-determined expression profile of putative switchgrass homologues to Cellulose Synthase. By assembling the switchgrass ESTs sequences with significant similarity to *OsCesA* genes, a set of seven contigs were obtained representing potential unique members of the *SGCesA* family. Further deduced protein sequence analysis of the *SGCesA* revealed that they contain the characteristic domain structure of CesA proteins [37], [38] that includes four conserved regions (U1, U2, U3 and U4), each containing a D residue or QXXRW sequence (D-D-D-QXXRW motif). The hypervariable region HVR-II located between the U2 and U3 which is found only in plant CesAs is also present (File S2). Gene-specific primers located in the hypervariable region HVR-II were designed to analyze expression patterns of the identified *SGCesA* genes.

**Figure 4:**
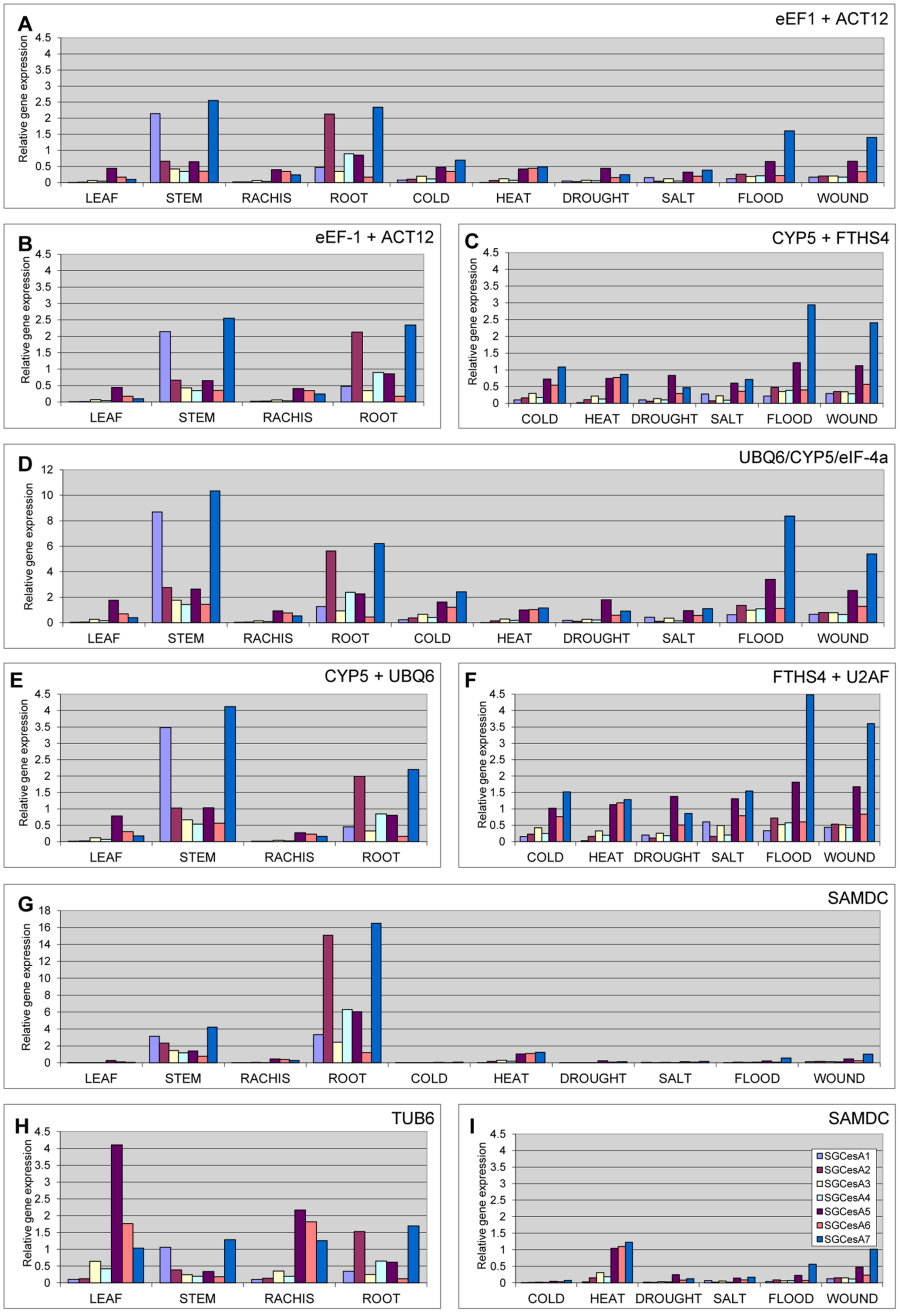
Relative quantification of SGCesAs genes using different combinations of reference genes for normalization. **A**. Gene expression normalized with the best combination of reference genes selected by NormFinder for all set of samples under study. **B**. Gene expression normalized with the best combination of reference genes selected by NormFinder for tissue samples. **C**. Gene expression normalized with the best combination of reference genes selected by NormFinder for samples under abiotic stress. **D**. Gene expression normalized with the most stable reference genes selected by geNorm for all samples under study. **E**. Gene expression normalized with the most stable reference genes selected by geNorm for tissue samples. **F**. Gene expression normalized with the most stable reference genes selected by geNorm for samples under abiotic stress. **G**. Gene expression normalized with the less stable genes identify by both algorithms for all samples under study. **H**. Gene expression normalized with the less stable genes identify by both algorithms for tissue samples. **I**. Gene expression normalized with the less stable genes identify by both algorithms for samples under abiotic stress.

To confirm and compare the results from the *SGCesAs* expression, different combinations of reference genes were used for normalization. When the set of samples from different tissues was analyzed, normalization was accomplished by using the geometric mean of *eEF-1α* and *ACT12* or *UBQ6* and *CPY5*, which are the best combination of genes determined by NormFinder and geNorm respectively. For the set of samples subjected to abiotic stress, the expression normalization was performed by using *CYP5* and *FTHS4* (NormFinder) or *U2AF* and *FTHS4* (geNorm). When all of samples under study were analyzed, normalization was calculated by using *eEF-1α* and *ACT12*, determined by NormFinder or *UBQ6*, *CYP5* and *eIF-4a*, the three most stable genes identify by geNorm and recommended by this program after the pairwise variation analysis (Figure 3B). As previously observed in other plant species [39]–[42], the expression levels for the *SGCesA* family members show variation among different tissues and abiotic stress conditions. The results reveal that the expression level of *SGCesA1* and *SGCesA7* was the highest in the stems. *SGCesA7* was also highly expressed in roots along with *SGCesA2*. On leaves and rachis a low transcript expression was detected for all the *SGCesAs* genes (Figure 4a, b, d, e). Among the leaf samples under abiotic stress conditions, expression analysis show that, in most of the tissues, *SGCesA5*, *6* and *7* transcript levels are higher than the other genes. Especially high is the level of expression of *SGCesA5* and *SGCesA7* in leaves under flood and wounding stress conditions (Figure 4a, c, d, f). A similar expression pattern was obtained when the best combination of genes identified by geNorm and NormFinder was used. However, a marked increase in the transcript abundance was observed in the samples normalized using the genes proposed by geNorm than the genes proposed by NormFinder (Figure 4a–f). Normalization based on the less stable genes show that the expression pattern varied considerably or the expression level was underestimated (Figure 4g–i).

Genes in the cellulose synthase (*CesA*) super family have been associated with the primary or secondary cell wall cellulose synthesis. Based on sequence similarities to the known *CesA* genes from Arabidopsis and other species [13], [43], [44] the identified *SGCesA2-7*, expressed in most of the tissues, are potentially involved in the primary wall cellulose synthesis. On the other hand, *SGCesA1*, show similarities to CesA involved in the secondary cell wall synthesis. This gene is highly expressed only in stems representing initiation of vascular development.

## Discussion

With the interest in improving switchgrass as a forage or bioenergy crop the need for the development of new tools for genomics has been emphasized [2], [45], [46]. Gene expression analyses could lead to a better understanding of processes involved in commercially-important traits in switchgrass. Quantitative Real Time PCR (qRT-PCR) provides an important tool for gene expression analysis. Although some studies on switchgrass gene expression have used polyubiquitin, actin, *eIF-4a*, *GAPDH* or *eEF-1α* as reference genes [7], [25]–[28] the validation of a suitable set of reference genes is of critical importance. The switchgrass transcriptome analysis and the development of a gene expression atlas (PviGEA) [16] allowed the identification of a group of stably expressed genes in samples of tissues under different stages of development. These resources are useful to select candidate reference genes for gene expression normalization in this specie but a validation of their expression stability is needed across experimental conditions and tissues.

The present work is the first detailed study on the stability of a set of genes that aims to identify a set of control genes for normalization of transcript levels in switchgrass. In this study, we selected a series of candidate reference genes for which sequence information was available in the switchgrass dbEST. After determination of primer amplification efficiencies, 11 candidates were selected for evaluation of their normalization potential along a group of 30 samples from different tissues and experimental conditions.

Since there is no universal accepted method, the comparison of two different computer programs, geNorm [22] and NormFinder [23], was used to select the best internal controls for normalization of gene expression studies in switchgrass. Differences were observed after comparison of the ranking of the candidate reference genes from these two programs (Figure 3, Table 3). This is an expected outcome due to the different mathematical approaches to calculate stability associated with each method [47]–[49]. Nevertheless, the expression stability analysis showed that six of the top seven most stable genes selected in each sample set were common for the two programs. Genes as *eEF-1α*, *e-IF-4a*, *CYP5* and *U2AF* are ranked by both algorithms among the most stable genes in the sample sets under study. The *eEF-1α* gene has been identified as a stable reference gene in other species including other members of the Poaceae family such as *Brachiaria* [50], lolium [35], perennial ryegrass [51] and rice [33]. Although less frequently, *eIF-4a* has been also identified as a good reference gene in some tissues as stems in flax [52] and in rice seeds [53]. The cyclophilins have been used in some reference genes studies where they have been identified usually as variable genes [52], [54], [55], however in our results *CYP5* is included among the most stable genes. A new reference gene identified in rice, *U2AF* [34], has been also reported as one of the most stably expressed gene in switchgrass, being an example of information transfer from a model plant to a crop. The ranking of the least stable genes determined by NormFinder was the same as in geNorm, both programs discard the use of *SAMDC* and *TUB6* because they display unacceptable expression stability limiting its use as internal control in *P.virgatum*. On the other hand, genes used previously as reference genes in switchgrass such as *eEF-1α* and *eIF-4a* [7], [27], are classified among the most stable genes by geNorm and NormFinder but other genes, as actin and *GAPDH* [25]–[28], show variations in the transcript levels in the set of leaf samples under abiotic stress conditions and leaf/stem, respectively. These results suggest that genes used commonly as reference genes should be used with caution requiring a careful evaluation for every set of samples under different experimental conditions before use.

Although the ranking of the different reference genes provided by both programs is similar, our results show that the genes that are most appropriate for use as reference genes can change, in the same set of samples and that each condition require a specific set of reference genes (Figure 3, Table 3). For tissue samples, geNorm recommended the use of *UBQ6* and *CYP5* and NormFinder recommended *eEF-1α* and *ACT12*. For leaves samples of plants under abiotic stress conditions *CYP5* and *FTHS4* were selected by geNorm and *U2AF* and *FTHS4* for NormFinder. For all set of samples under study, *eEF-1α* and *ACT12*, were determined by NormFinder and *UBQ6*, *CYP5* and *eIF-4a* by geNorm. To determine the effectiveness of selected reference genes, we evaluated their efficacy to normalize seven members of the *SGCesA* gene family in samples of different tissues and under different abiotic stress conditions (Figure 4). The quantification of *SGCesA* using different control genes showed that the choice of the internal standard is very important in the normalization of the target gene expression levels. Similar expression patterns were generated when the best combination of genes selected by both programs was used but differences were detected in the transcript abundance of the target genes (Figure 4 a–f). This is due to the highest transcript level of the reference genes selected by NormFinder compare with the genes selected by geNorm.

To quantifying expression, data analysis was performed by a relative approach developed in DART-PCR [29]. The starting fluorescence (R_0_), which is proportional to the starting template quantity, is calculated taking in account the threshold cycle (Ct), the fluorescence at this cycle (R_Ct_) and the amplification efficiency (E), based on the equation: R_0_ = R_Ct_×(1+E)^−Ct^. The R_0_ of each sample was normalized by the normalization factor obtained as the geometric mean of the reference genes R_0_ values. Any difference in the Ct, E and R_Ct_ values is able to produce differences on the resulting R_0_ value and consequently, on the normalized expression values. As an example, we compare the normalized samples subjected to flooding conditions. Despite the similar Ct values of the reference genes selected for normalization by NormFinder (*CYP5*, 23.88 and *FTHS4*, 22.81) and geNorm (*U2AF*, 24.16 and *FTHS4* 22.81) the normalization factors R_0_ values were 1.24×10^−8^ and 8.50×10^−9^, respectively. The use of these NFs results in a difference of approx. 30% between the relative gene expressions of the genes (Figure 4 c, f). We consider that the genes selected by both programs are valid for normalization but when the transcript abundance of the gene under study is low, reference genes with also low abundance will improve the measurement accuracy. Next we used the genes identified by both programs with the lowest expression stability for normalization (Figure 4g–i). Expression patterns varied considerably in comparison with the result from the use of the most stable genes. For example, the low expression level of *TUB6* in leaf samples leads to an overestimation of *SGCesA5* (Figure 4h) and the high transcript level of *SAMDC* gives an underestimation of the SGCesAs in samples under abiotic stress were the transcript abundance is undetectable in some samples (Figure 4i). It suggested that not only the stability but also the abundance of a reference gene affect the normalized results and demonstrate the need to evaluate the stability of the reference genes before to be used in a set of samples.

## Conclusions

To the best of our knowledge, this work is the first study aimed to validate a set of candidate reference genes for gene expression normalization using qRT-PCR in switchgrass. In the present research 11 candidate reference genes were identified in the EST database of switchgrass and their expression stability was evaluated across a set of 30 samples using the computer programs geNorm and NormFinder. The use of the reference genes selected by both programs to determine the expression profile of the target genes allowed us to determine the most adequate combination of control genes. These results may constitute a starting point to select reference genes in the future to more accurate normalization in other tissues and under other experimental conditions in switchgrass.

## Supporting Information

File S1 **SGCesA partial sequences.** Consensus sequences after Switchgrass ESTs assembly. In gray gene specific primer in the HVR-II of the SGCesAs.
(PDF)

File S2 **Alignment of the partial-length deduced amino acid sequence of putative** ***P. virgatum*** **cellulose synthase proteins.** Amino acid sequence alignment of the highly conserved U1, U2 and U3/4 regions of the predicted SGCesAs. Domains are indicated below the sequence as follows: shaded amino acid with light gray are U domains, the D, D, D, QXXRW motif is indicated in red, the conserved region is underlined with light gray shading within a box, and the variable region (HVR-II) with dark gray shading without a box.
(PDF)

Table S1 **Primer sequence, amplicon and efficiency of the** ***SGCesA*** **genes evaluated in this study.**
(PDF)
