# Supplementary material for: Selection and Validation of Reference Genes for Gene Expression Analysis in Switchgrass (Panicum virgatum) Using Quantitative Real-Time RT-PCR
Source: PLoS One. 2014 Mar 12;9(3):e91474. doi: 10.1371/journal.pone.0091474 (PMC3951385; doi:10.1371/journal.pone.0091474)
Supplement: File S1 — SGCesA partial sequences. Consensus sequences after Switchgrass ESTs assembly. In gray gene specific primer in the HVR-II of the SGCesAs. (PDF) [file pone.0091474.s001.pdf]

>SGCesA1
GCTGAAGCGGACGAGGTGGAGGACCACCACGCCGGCGGCGGCCTGCGTGAGAGGGTCACCATGGGCAGCCACCTCAATGATCGCCAGGATGAAGTAAGCCA
TGGCAGGACCATGAGCAGCTTGTCGGGAATTGGTAGTGAACTGAATGATGAATCTGGAAAACCCATCTGGAAGAACAGGGTGGAGAGTTGGAAGGAGAAGA
AGAACGAGAAAAAAGCCTCAGCCAAAAAGGCTGCAGCAAAAGCACAGGCACCACCTGTCGAAGAACAGATCATGGATGAAAAGGATTTGACAGATGCGTATG
AACCGCTCTCCCGGATCATCCCAATATCAAAGAACAAGCTTACACCTTACAGAGCGGTCATCATTATGCGTTTGATAGTTCTTGGGCTCTTCTTTCACTACCGTATC
ACCAATCCTGTTTACAGTGCCTTTGGTCTCTGGATGACATCAGTCATATGTGAGATCTGGTTTGGTTTCTCCTGGATCTTGGATCAATTCCCCAAGTGGTACCCTA
TTAATCGAGAGACTTATGTTGATAGGCTGATTGCAAGATACGGAGATGGTGAAGATTCTGGATTGGCACCTGTGGATTTCTTTGTCAGTACAGTGGATCCATTG
AAAGAGCCTCCACTGATCACTGCAAACACTGTATTGTCTATTCTTGCTGTGGACTATCCTGTTGAGAAGATCTCATGCTATGTATCTGATGATGGTTCTGCTATGC
TCACATTTGAATCACTTGCCGAGACTGCTGAATTTGCTAGAAAATGGGTACCATTTTGCAAGAAGTATGCGATTGAGCCACGAGCTCCTGAGTTCTATTTCTCGC
AGAAGATTGACTACTTGAAGGACAAGATACACCCGTCTTTTGTCAAGGAGCGTAGGGCTATGAAGAGAGACTATGAAGAATACAAGGTGAGGATAAATGCTTT
GGTCGCCAAGGCTCAGAAGACACCTGACGAAGGCTGGATCATGCAAGACGGTACACCATGGCCTGGGAATAATCCTCGTGACCACCCTGGCATGATCCAGGTT
TTCCTTGGTGAGACTGGTGCCCGGGACTTTGATGGTAACGAACTTCCTCGTTTAGTGTATGTTTCAAGAGAAAAAAGACCAGGCTACCAACACCACAAGAAGGC
TGGGGCCATGAATGCTCTGGTCCGAGTGTCTGCTGTCCTGACAAATGCTCCTTACATTCTTAATCTTGATTGTGATCACTATGTTAACAACAGCAAAGCCGTCCGT
GAAGCAATGTGCTTCATGATGGACCCTACTGTTGGCAGAGATGTCTGCTATGTACAATTCCCACAGAGGTTCGATGGCATTGATCGCAGTGATCGATACGCCAA
TAGGAATGTTGTGTTCTTTGACGTTAACATGAAAGGACTTGATGGCATTCAAGGCCCCGTTTATGTGGGAACTGGTTGTTGTTTCTATAGGCAAGCACTGTATGG
TTATGGACCTCCATCTCTGCCTGCCCTCCCAAAATCTTCGATTTGTTCGTGGTGTTGCTGTTGCTGTCCCAAGAAGAAGGCTGAAAGAAGTGAGAGGGAAATCAA
CAGGGACTCTCGGCGAGAAGACCTTGAGTCTGCCATTTTCAATCTTCGGGAAATTGACAACTACGATGAGTATGAGAGGTCCATGCTTATCTCTCAGATGAGCTT
TGAGAAGACTTTTGGCCTGTCCTCAGTATTCATTGAATCAACCCTTATGGAGAATGGGGGCGTCCCTGAATCTGCAAACCCATCTACTCTCATTAAAGAAGCCAT
TCATGTCATTAGCTGTGGATATGAAGAGAAAAGTGAATGGGGAAAAGAGATTGGCTGGATTTACGGTTCAGTTACAGAGGATATTCTGACTGGGTTCAAGATG
CACTGCCGTGGCTGGAGATCCATCTACTGCATGCCGGTGAGACCTGCATTCAAGGGATCAGCGCCTATCAATCTTTCCGATCGTCTGCACCAGGTTCTCCGGTGG
GCTCTTGGTTCCGTTGAGATCTTCCTGAGTCGGCACTGCCCGCTGTGGTATGGTTACGGTGGTGGCCGTCTGAAATGGCTTCAGAGGCTATCCTACATCAACACC
ATCGTCTACCCGTTCACTTCTCTTCCTCTTATTGCCTACTGTTGCCTGCCTGCCATTTGCCTGCTCACCGGAAAGTTCATCATTCCAACGCTGTCCAACGCCGCGAC
AATATGGTTTCTTGGCCTCTTCATGTCCATCATCTTGACGAGCGTGTTGGAGCTGCGGTGGAGTGGCATCGGCATCGAGGACTGGTGGCGCAACGAGCAGTTCT
GGGTCATCGGAGGCGTGTCCGCGCACCTGTTCGCCGTGTTCCAGGGTATCCTCAAGATGATTGCCGGGCTGGACACCAACTTCACGGTCACGGCCAAGGCGAC
GGACGACACCGAGTTCGGCGAGCTGTACGTGTTCAAGTGGACGACGGTGCTGATCCCGCCGACCACCATCCTGGTGCTCAACATGGTGGGCGTGGTGGCCGG
GTTCTCCGACGCGCTCAACAGCGGCTACGAGTCGTGGGGCCCGCTCTTCGGCAAGGTGTTCTTCGCCATGTGGGTGATCATGCACCTGTACCCGTTCCTCAAGG
GTCTCATGGGCCGCCAGAACCGCACGCCCACCATCGTCGTGCTCTGGTCCGTCCTGCTCGCTCCGTCTTCTCCCTCCTCTGGGTCAAGATCGACCCCTTCATCGGA
GGCACCGAGCCTATCAACAGCACCAACTGCAACACAGTCATCTGCTGAGGGCCGCCGTCA

>SGCesA2
GCGGCGGCGGCGGCGGGGGCAAGAGGATCCACCCGCTCCCCTTCGCAGATCCGAGCCTTCCAGTGCAACCGAGATCCATGGACCCGTCCAAGGATCTGGCCGC
CTACGGCTACGGCAGCGTGGCCTGGAGCGACCGGATGGAGCGCTGGAAGAAGAACCAGGAGAGCCTGCAGCACGCCAGGAGCGACGGCGGCGGTGATTGG
GACGGCGACGATGCAGATCTGCCGCTAATGGATGAAGCAAGACAGCCATTGTCCAGAAAAGTTCCGATACCATCAAGCCGAATTAACCCCTACAGGATGATTAT
TGTTATCCGGTTGGTGGTTTTGGGGTTCTTCTTCCACTACCGTGTGATGCATCCGGTGAATGATGCATTTGCTTTATGGCTCATATCTGTAATCTGTGAAATCTGG
TTTGCCATGTCCTGGATTCTTGATCAGTTCCCAAAGTGGCTTCCAATTGAAAGAGAGACTTACCTGGACCGTTTGTCACTAAGATTTGACAAGGAAGGCCGACCC
TCTCAACTTGCTCCAGTTGACTTCTTTGTCAGTACGGTTGATCCCTCAAAGGAGCCTCCCTTGGTCACAGCAAACACTGTCCTTTCCATCCTTGCTGTTGATTATCC
AGTTGAGAAGGTCTCCTGCTATGTTTCTGATGATGGTGCTGCAATGCTTACATTTGAAGCACTGTCTGAAACATCCGAATTTGCAAAGAAATGGGTTCCTTTCTG
CAAAAAGTTTAATATTGAGCCTCGTGCTCCTGAGTGGTACTTCCAACAGAGATAGACTACCTGAAAGACAAGGTTGCCGCATCCTTTGTTAGGGACAGGAGGGC
AATGAAGAGGGAATATGAGGAATTCAAGGTAAGAATCAATGCCTTGGTTGCGAAAGCCCAAAAGGTTCCCGAGGAAGGATGGACAATGCAGGATGGAAGCCC
CTGGCCTGGAAACAATGTACGCGATCATCCTGGAATGATTCAGGTATTCCTTGGCCAAAGCGGTGGTCATGATGTGGAAGGAAATGAGCTGCCTCGCCTCGTTT
ATGTCTCGAGAGAAAAGAGGCCAGGCTATAACCATCATAAGAAGGCTGGTGCCATGAATGCACTGGTTCGTGTCTCTGCTGTCTTATCAAATGCTCCATACCTAT
TGAACTTGGACTGTGATCACTACATCAACAATAGCAAGGCCATAAAAGAGGCTATGTGTTTCATGATGGATCCTTTGGTGGGGAAGAAAGTGTGCTACGTGCAG
TTCCCTCAGAGGTTTGATGGTATTGACCGGCATGATCGATACGCTAACAGGAACGTTGTCTTCTTTGATATCAACATGAAAGGTCTGGATGGTATTCAAGGACCC
ATCTATGTGGGTACTGGATGTGTCTTTAGACGGCAGGCACTGTATGGTTATGATGCTCCGAAAACCAAGAAGCCACCATCAAGAACTTGCAATTGCTGGCCCAA
GTGGTGCCTCTCTTGCTTCTGCTGCAGAAACAAGAGTAAGAAGAAGACTACAAAACCGAAAACAGAGAAGAAGAAGAAAAGATTATTTTTCAAGAAAGCAGAA
AATCCATCTCCTGCATACGCCCTGGGTGAAATTGAGGAAGGTGCTCCAGGTGCTGACGTTGAGAAGGCTGGAATTGTAAATCAACAGAAGCTTGAAAAGAAGT
TCGGGCAATCTTCTGTATTTGTTGCATCAACACTTCTTGAGAATGGTGGGACCTTGAAGAGTGCAAGTCCAGCATCGCTACTGAAGGAAGCTATACATGTTATCA
GCTGCGGTTATGAAGACAAGACTGACTGGGGGAAAGAGATTGGCTGGATTTATGGATCAATTACAGAAGATATCTTGACTGGATTTAAGATGCACTGCCATGG
TTGGCGGTCTATTTATTGCATCCCAAAGAGGCCTGCATTCAAAGGTTCTGCACCTCTGAATCTTTCTGATCGTCTTCACCAGGTCCTTCGGTGGGCTCTTGGATCT
GTTGAAATTTTCTTCAGCAAGCACTGCCCACTTTGGTATGGGTATGGTGGTGGGCTAAAATTTTTGGAGAGGTTCTCATATATTAACTCTATCGTATACCCCTGGA
CATCCATTCCGCTGTTGGCTTACTGTACATTGCCTGCCATATGCTTGCTCACGGGGAAATTTATCACACCAGAGCTTACCAATGCTGCCAGTATCTGGTTCATGGC
GCTTTTCATCTGCATCTTTATCACCGGCATACTTGAAATGAGATGGAGTGGTGTGGCCATCGACGACTGGTGGAGAAACGAGCAATTCTGGGTCATTGGAGGTG
TTTCCTCACATCTCTTCGCCGTGTTCCAAGGCCTCCTGAAGGTCCTCGCCGGTATCGACACCAGCTTCACCGTGACATCGAAGGGCGGGGACGACGAGGAGTTCT
CGGAGCTGTACACCTTCAAATGGACGACCCTGCTGATCCCGCCGACGACGCTGCTCCTGCTGAACTTCATCGGGGTCGTGGCCGGGATCTCCAACGCGATCAAC
AACGGGTACGAGTCGTGGGGCCCCCTGTTCGGGAAGCTCTTCTTCGCCTTCTGGGTGATCGTCCACCTGTACCCGTTCCTCAAGGGTCTGGTGGGGAGGCAGAA
CCGGACGCCGACGATTGTCATCGTCTGGTCCATCCTGCTGGCCTCCATCTTCTCGCTCCTGTGGGTGCGCATCGACCCGTTCCTCGCCAAGAGCGACGGCCCCCT
CCTGGAGGAGTGCGGCCTGGACTGCAACTGAGGAAGAAAGAGGAAGACCCCTCCTGGCGCAGGCGAATTACGCCCTGAATTTTTTGGAATTCTTTCCTGTAGA
TAGAAACACAGATCCTCCCCTCCTTCCGGCGCTCCTTCTGTTCCATGGCGGCGAGGCCGTGACATCCCTGTGCAGCTCTTCTTCAACATTCCCCATTCATTCATCCG
AGGAACCCGCACCGGATTGATCCGCT

>SGCesA3
GGATGCACCAGATGAGGGACGAAGGAGGTGGCAACGATGATGGTGATGATGCAGATCTACCACTAATGGATGAAGCTAGACAGCCATTGTCCAGAAAGATTC
CACTTCCTTCAAGCCAGATCAATCCCTATAGAATGATTATCATAATTCGACTAGTGGTTTTGGGGTTCTTCTTCCACTATCGAGTGATGCATCCGGTGCCTGATGC
ATTTGCTTTATGGCTTATATCTGTCATCTGTGAGATATGGTTTGCCATGTCTTGGATTCTTGATCAGTTCCCAAAGTGGTTTCCTATTGAGAGGGAAACCTATCTTG
ACCGGTTGACTTTGAGGTTTGACAAGGAAGGGCAGCCTTCTCAACTCGCCCCAGTCGATTTCTTTGTCAGTACAGTTGATCCCATGAAGGAACCTCCGTTGGTCA
CAGCAAATACTGTTCTATCTATCCTGGCAGTGGATTATCCAGTTGATAAGGTTTCATGCTATGTTTCTGATGATGGTGCTGCCATGCTGACATTTGAAGCATTGTC

TGAAACATCTGAATTTGCAAAGAAATGGGTTCCTTTCTGCAAAAAATACAGTATTGAGCCTCGTGCTCCAGAATGGTACTTCCAACAGAAGATAGACTACCTGAG
AGATAAGGTGGCTACAAACTTCGTTAGGGAGCGGAGAGCAATGAAGAGAGAGTATGAGGAATTCAAGGTCAGAATCAATGCCTTGGTTGCTAAAGCCCAAAA
GGTTCCTGAGGAAGGATGGACAATGCAGGATGGGACTCCATGGCCTGGAAACAACGTTCGTGATCATCCTGGAATGATTCAGGTGTTCCTTGGTCAAAGTGGT
GGCCATGATGTGGAAGGAAATGAGCTGCCTCGATTGGTTTACGTTTCAAGAGAAAAACGGCCTGGCTACAACCATCATAAGAAGGCTGGTGCTATGAATGCAT
TGGTCCGAGTCTCTGCTGTACTAACCAATGCACCATATATGCTGAACTTGGATTGCGATCACTACATCAATAACAGCAAGGCTATTAAGGAAGCAATGTGTTTTA
TGATGGATCCTTTGCTAGGAAAGAAAGTTTGCTACGTGCAGTTTCCTCAAAGGTTCGATGGGATTGATCGTCATGATCGATATGCCAACAGGAATGTTGTCTTTT
TCGATATCAACATGAAAGGCTTGGATGGTATTCAAGGCCCCATATACGTCGGTACTGGATGCGTCTTCAGAAGGCAGGCATTATATGGCTACGATGCCCCCAAA
ACAAAGAAGCCACCATCAAGGACTTGCAACTGCTGGCCAAAGTGGTGATTTGCTGTTGCTGTTTTGGTAACAGGAAGACCAAGAAGAAGACCAAGAGATCAAA
GCCTAAATTTGAGAAGATAAAGAAGCTTTTCAAGAAAAAGGAAAATCAAGCCCCTGCGTATGCTCTTGGTGAAATTGACGAAGCTGCTCCAGGAGCGGAAAAT
GAAAAGGCTAGTATTGTAAATCAACAGAAGTTAGAAAAGAAATTTGGCCAGTCTTCAGTTTTTGTTGCTTCCACACTTCTTGAGAATGGTGGTACCCTGAAGAGT
GCCAGTCCAGCTTCTCTTCTAAAGGAAGCTATACATGTCATCAGTTGTGGCTATGAGGACAAGACAGACTGGGGGAAAGATATTGGTTGGATTTATGGATCAGT
TACAGAAGATATTCTTACTGGGTTTAAGATGCACTGCCATGGTTGGCGATCAATTTACTGCATACCTAAATGGGCCGCCTTCAAAGGTTCCGCACCTCTCAATCTT
TCTGATCGTCTTCACCAGGTTCTTCGGTGGGCTCTTGGTTCGATCGAAATTTTCTTCAGCAACCATTGCCCTCTTTGGTATGGATATGGTGGTGGACTAAAATTTC
TGGAAAGGTTTTCCTACATTAACTCCATCGTATACCCGTGGACATCCATCCCGCTCTTGGCCTATTGCACATTGCCTGCTATCTGCTTGCTGACAGGGAAATTTATT
ACGCCAGAGCTTGACAATGTTGCCAGCCTCTGGTTCATGTCACTTTTTATCTGCATTTTTGCTACGGGTATCCTGGAAATGAGATGGAGTGGTGTAGGCATCGAT
GATTGGTGGAGAAATGAGCAGTTTTGGGTCATTGGAGGAGTGTCTTCGCATCTCTTTGCCGTGTTCCAAGGACTTCTCAAGGTCATAGCTGGTATAGATACGAG
CTTCACTGTGACGTCAAAGGGTGGAGATGATGAGGAGTTCTCGGAGCTGTACACATTCAAATGGACTACCCTTCTGATACCTCCAACCACATTGCTTCTCTTGAA
CTTCATTGGAGTGGTGGCTGGTGTTTCTAATGCAATAAACAACGGATATGAATCCTGGGGTCCCCTCTTTGGGAAGCTCTTCTTTGCATTTTGGGTGATGTCCATC
TGTACCCGTTCCTCAAGGGTTTGGT
>SGCesA4
CAGAAAGGTAGCCCTCGAGTTCACGGTGATGAGGAGGAAGAAGATGTTGATGACATTGACAATGAATTCAACTACGAGCAAGGCAATGGGAAAGGACCAGGG
TGGCAGCTGCATGGTCAGGGAGATGATGCTGATCTTTCTTCATCTGCTCGCCATGAGCCGCATCATCGAATTCCCCGCTTGACAAGTGGGCAGCAGATGTCTGG
AGAGATCCCTGATGCTTCCCCTGACCGTCATTCTATCCGCAGTCCAACATCAAGCTATGTTGATCCAAGCGTCCCAGTTCCCGTGAGGATTGTGGATCCCTCGAA
GGACTTGAATTCCTATGGGCTTAATAGTGTTGACTGGAAGGAAAGAGTTGAGAGCTGGAGGGTTAAACAGGATAAAAATATGATGCAAGTGACTAATAAATAT
CCAGAAGCTAGAGGAGACATGGAGGGAACTGGCTCAAATGGTGAAGATATGCAAATGGTTGATGACGCACGGCTACCTCTGAGCCGTATTGTGCCGATTCCCT
CAAACCAACTCAACCTTTACCGGATAGTGATCATTCTCCGTCTTATCATCCTGTGCTTCTTCTTCCAGTATCGTGTCACTCATCCAGTGCGTGATGCTTATGGATTG
TGGCTAGTATCTGTTATCTGTGAGGTTTGGTTTGCCTTGTCTTGGCTTCTTGATCAGTTCCCAAAATGGCATCCAATCAACCGTGAGACATACCTTGACAGGCTTG
CATTGAGGTATGATAGAGAGGGAGAGCCATCACAGCTGGCTCCCATTGATGTCTTTGTCAGTACAGTGGATCCATTGAAGGAACCTCCATTGATCACAGCCAAT
ACTGTTTTGTCCATACTTGCTGTGGATTATCCTGTTGACAAAGTGTCATGCTATGTTTCTGATGATGGCTCAGCTATGTTGACTTTTGAGGCTCTCTCAGAAACTGC
GGAATTTGCTAGGAAGTGGGTTCCCTTCTGCAAAAAGCACAACATTGAACCGAGAGCTCCTGAATTTTACTTTGCTCAAAAAATAGATTACCTAAAGGACAAAAT
CCAACCTTCATTTGTTAAGGAAAGGCGAGCAATGAAGCGAGAGTACGAAGAATTCAAAGTAAGAATCAATGCCCTTGTTGCCAAAGCACAGAAAGTGCCTGAA
GAGGGTTGGACCATGGCTGATGGAACTGCTTGGCCTGGGAATAATACAAGGGATCATCCTGGCATGATTCAGGTGTTCCTGGGGCACAGTGGTGGGCTTGACA
CTGATGGAAATGAGTTACCCCGCCTTGTCTATGTCTCTCGTGAAAAGAGACCAGGCTTCCAGCATCACAAGAAGGCTGGTGCAATGAATGCACTGATTCGTGTG
TCTGCTGTGCTAACAAATGGTGCCTATCTTCTCAATGTGGATTGTGACCATTATTTCAACAGCAGCAAAGCTCTTAGAGAAGCAATGTGCTTCATGATGGATCCT
GCACTAGGAAGGAAAACTTGTTACGTACAGTTTCCACAAAGATTCGATGGCATTGACTTGCATGATCGATATGCTAATCGCAACATAGTTTTCTTTGATATCAAC
ATGAAAGGTCTAGATGGCATCCAGGGTCCAGTTTATGTGGGAACAGGATGCTGTTTCAATAGGCAGGCTTTGTACGGCTATGATCCTGTATTGACCGAAGCCGA
TCTGGAACCTAACATTGTTGTTAAGAGCTGCTGTGGTAGAAGAAAGAAGAAGAACAAGAGTTACATGGATAGCCAAAGCCGTATTATGAAGAGAACAGAATCT
TCGGCTCCCATCTTCAACATGGAAGACATCGAAGAGGGTATTGAAGGTTATGAGGATGAAAGGTCAGTGCTTATGTCCCAGAGGAAATTGGAGAAACGCTTTG
GCCAGTCCCCAATTTTCATTGCATCCACCTTTATGACTCAAGGCGGCATACCACCTTCAACAAACCCAGCATCTCTACTAAAGGAAGCTATTCATGTCATCAGCTG
TGGCTACGAGGACAAAACTGAATGGGGCAAAGAGATTGGCTGGATCTACGGTTCAGTTACTGAGGATATTTTGACTGGGTTTAAAATGCACGCAAGAGGCTGG
CAGTCAATCTACTGCATGCCACCAAGGCCTTGTTTCAAGGGTTCTGCACCAATCAATCTTTCTGATCGTCTTAATCAGGTGCTCCGTTGGGCTCTTGGGTCAGTTG
AAATTCTGCTTAGCAGACATTGTCCTATCTGGTATGGCTACAATGGGCGGTTGAAGCTTCTGGAGAGGCTGGCTTACATCAACACCATCGTTTATCCAATCACAT
CCATTCCACTTATCGCCTATTGTGTGCTTCCTGCTATCTGTCTCCTTACCAATAAATTTATCATTCCTGAGATCAGTAATTATGCTGGGATGTTCTTCATTCTTCTTTT
CGCCTCCATTTTTGCTACTGGTATATTGGAGCTCAGATGGAGTGGTGTTGGCATTGAAGATTGGTGGAGAAATGAACAGTTTTGGGTTATTGGTGGCACCTCTG
CCCATCTCTTCGCCGTGTTCCAGGGTCTGCTGAAAGTGTTGGCTGGGATCGATACCAACTTCACAGTTACCTCAAAGGCATCTGATGAAGATGGCGACTTTGCTG
AGCTTTATGTGTTCAAGTGGACCAGTTTGCTCATCCCGCCAACCACTGTCCTTGTCATTAACCTGGTTGGAATGGTGGCAGGGATTTCATATGCCATTAACAGCG
GCTACCAATCCTGGGGTCCGCTTTTTGGGAAGCTGTTCTTCTCAATCTGGGTGATCCTCCATCTCTACCCCTTCCTCAAGGGTCTCATGGGCAGGCAGAACCGCAC
ACCAACCATTGTCATTGTCTGGTCCATTCTCCTTGCGTCCATCTTCTCTCTGTTGTGGGTGAAGATCGACCCTTTCATCTCCCCAACACAGAGAGCTGTTGCTTTGG
GGCAATGCGGCGTGAACTGCTGATCAGAC

>SGCesA5
AGGGGATGTTGGCCGCCCCAAGTATGACAGTGGCGAGATCGGGCTCTCCAAGTATGACAGTGGTGAGATCCCTCGAGGCTACATCCCGTCAGTCACAAACAGC
CAGATCTCGGGAGAAATTCCTGGAGCTTCCCCTGACCATCATATGATGTCCCCTACTGGGAACATTGGCAAGCGCGCTTCGTTTCCCTATGTGAATCATTCGCCA
AATCCATCACGGGAGTTCTCTGGTAGCATTGGAAATGTTGCCTGGAAAGAGAGAGTTGATGGCTGGAAAATGAAGCAGGATAAAGGAACAATTCCCATGACTA
ATGGCACCAGCATTGCTCCCTCTGAGGGTCGGGGTGTTGGTGACATTGATGCATCTACTGATTACAACATGGAAGACGCCTTACTGAACGATGAAACTCGACAG
CCTCTATCCAGAAAAGTGCCAATTCCTTCCTCCAGGATCAATCCTTACAGAATGGTCATTGTGCTGCGATTGGTTGTTCTAAGCATCTTCCTGCACTATCGTATCAC
AAATCCTGTGCGCAATGCATACCCGTTATGGCTTCTATCTGTTATATGTGAGATTTGGTTTGCTCTTTCCTGGATATTAGATCAGTTCCCAAAGTGGTTTCCAATCA
ACCGTGAGACTTACCTTGATAGATTGGCATTAAGGTATGACCGTGAAGGTGAGCCATCTCAGTTGGCTGCTGTTGACATCTTTGTCAGTACAGTCGACCCAATGA
AGGAGCCACCTCTTGTCACTGCCAATACTGTTCTATCAATTCTTGCTGTGGATTACCCTGTCGATAAGGTCTCTTGCTATGTATCTGACGATGGAGCTGCAATGCT
GACATTTGATGCACTAGCTGAGACTTCAGAATTTGCTAGAAAATGGGTACCGTTTGTTAAGAAGTACAACATTGAGCCTAGAGCTCCTGAATGGTACTTCAGCCA
GAAAATTGATTACTTGAAGGACAAAGTCCACCCTTCATTTGTTAAAGACCGCCGTGCCATGAAGAGAGAATATGAAGAATTTAAAATTAGGATAAATGGCCTTG
TTGCTAAGGCACAGAAAGTTCCTGAGGAAGGATGGATCATGCAAGATGGCACACCATGGCCAGGAAACAATACCAGGGACCATCCTGGAATGATTCAGGTTTT
CCTTGGTCACAGTGGTGGCCTTGATACTGAGGGCAATGAGCTTCCCCGTTTGGTCTATGTTTCTCGTGAGAAGCGTCCTGGATTCCAACATCACAAGAAAGCTGG
TGCCATGAATGCTCTTGTTCGTGTGTCAGCTGTGCTTACCAATGGACAATACATGTTGAATCTTGACTGTGATCACTACATCAACAACAGCAAGGCTCTCAGGGA
AGCTATGTGCTTCCTTATGGATCCTAACCTAGGAAGGAGTGTCTGCTATGTTCAGTTTCCACAAAGGTTCGATGGTATTGATAGGAACGATCGATATGCCAACAG
GAACACCGTGTTTTTCGATATTAACTTGAGGGGTCTTGATGGCATTCAAGGACCAGTTTATGTGGGAACTGGTTGTGTTTTCAACAGAACAGCTCTATATGGTTA

TGAGCCCCCAATTAAGCAAAAGAAGAAGGGTGGTTTCTTGTCATCACTATGTGGTGGCAGGAAGAAGGCAAGCAAATCCAAGAAGGGCTCAGACAAGAAAAA
GTCACAGAAGCATGTGGACAGTTCCGTGCCAGTATTCAATCTTGAAGATATAGAGGAGGGAGTTGAAGGTGCTGGATTTGATGATGAGAAATCACTTCTTATGT
CTCAAATGAGCTTGGAGAAGAGATTTGGCCAATCTGCAGCTTTTGTTGCCTCCACACTGATGGAATATGGTGGTGTTCCTCAGTCTGCAACTCCAGAATCTCTTCT
GAAAGAAGCTATTCATGTCATAAGTTGTGGCTATGAGGATAAGACTGAATGGGGAACTGAGATTGGGTGGATCTATGGTTCTGTGACAGAAGATATTCTTACTG
GATTCAAGATGCATGCACGAGGCTGGCGGTCAATTTACTGCATGCCTAAGCGGCCAGCTTTCAAGGGATCTGCTCCCATCAATCTTTCAGACCGTCTGAACCAAG
TGCTCCGGTGGGCTCTTGGTTCTGTGGAAATTCTTTTCAGCCGGCATTGCCCCTTATGGTATGGCTATGGAGGACGCCTGAAGTTCTTGGAGAGATTTGCTTACA
TCAACACCACCATTTACCCACTCACGTCTATCCCACTTCTCATATACTGTGTTCTGCCTGCCATCTGTCTGCTCACTGGGAAGTTCATCATCCCAGAGATTAGCAAC
TTCGCTAGTATTTGGTTCATCTCTCTCTTTCTTTCAATTTTTGCCACTGGTATCCTCGAGATGAGGTGGAGTGGTGTTGGCATTGATGAGTGGTGGAGGAATGAAC
AATTCTGGGTTATTGGAGGTATCTCTGCCCATCTTTTCGCCGTATTCCAGGGTCTTCTCAAGGTGCTTGCTGGTATCGACACCAACTTTACTGTCACATCAAAGGC
CTCTGATGAAGATGGTGACTTCGCGGAGCTCTACATGTTCAAGTGGACAACTCTTCTCATCCCACCAACAACCATCTTGATCATCAACCTGGTCGGTGTTGTTGCT
GGTACCTCCTATGCTATCAACAGCGGTTACCAATCATGGGGGCCACTCTTTGGCAAGCTCTTCTTCGCCTTCTGGGTGATTGTTCACTTGTACCCATTC

>SGCesA6
GGCACACAGGCGTGCCCGCAGTGCAAGACCAAGTACAAGCGCCACAAGGGGAGCCCTCCTGTGCATGGTGAGGAAAATGAGGATGTTGACGCGGACGATGTG
AGTGACTACAACTACCCAGCATCTGGCAACCAGGATCAGAAGCAGAAGATTGCTGAGAGAATGCTCACCTGGCGCACCAACTCACGGGGTAGCGATGTTGGCC
TTGCTAAGTATGACAGCGGTGAGATTGGGCATGGGAAGTATGACAGTGGTGAGATCCCTCGAGGATATATCCCGTCACTCACTCATAGCCAGATCTCTGGAGA
AATTCCTGGAGCTTCCCCTGATCATATGATGTCTCCTGTTGGGAACATTGGCAGGCGTGGACATCAATTTCCCTATGTGAATCATTCTCCAAACCCATCAAGGGA
GTTCTCTGGTAGCCTTGGTAATGTTGCATGGAAAGAGAGAGTGGATGGATGGAAAATGAAGGACAAGGGTGCAATTCCTATGACTAATGGGACAAGCATTGCT
CCCTCAGAAGGACGTGGTGTTGGTGATATTGATGCATCAACTGACTATAACATGGAAGACGCTTTACTGAATGATGAAACTCGGCAACCTCTATCAAGAAAAGT
GCCAATTCCTTCATCCAGAATAAATCCCTACAGAATGGTCATCGTCCTACGTTTGATTGTTCTATGCATTTTCCTGCACTACCGTATCACAAATCCTGTGCGCAATG
CATATCCACTGTGGCTGTTGTCCGTTATATGTGAGATTTGGTTTGCTCTGTCCTGGATCTTGGATCAGTTCCCAAAGTGGTCCCCAATCAATCGTGAAACCTACCT
TGATAGACTGGCACTAAGGTATGACCGAGAAGGTGAACCATCTCAGTTGGCTCCTGTTGATATTTTTGTCAGTACCGTGGACCCTATGAAGGAGCCTCCTCTTGT
CACTGCAAATACTGTGCTTTCCATTCTTGCTGTGGACTATCCGGTTGACAAGGTATCTTGCTATGTGTCTGATGATGGAGCTGCTATGCTGACTTTTGATGCACTC
GCTGAAACTTCAGAGTTTGCTAGAAAATGGGTACCATTCTGTAAGAAGTACAACATAGAACCTAGGGCTCCAGAGTGGTACTTCGCTCAAAAAATTGATTACTT
GAAAGACAAAATCCAAACTTCATTTGTTAAAGACCGCCGGGCAATGAAGAGAGAATATGAAGAATTCAAAGTCCGTATTAATGGTCCTTGTAGCCAAAGCACAG
AAAGTTCCCGAGGAGGGATGGATCATGCAAGATGGTACACCTTGGCCTGGGAACAATACTAGGGACCATCCTGGAATGATTCAGGTTTTTCTAGGTCACAGTG
GAGGCCTTGATGCTGAGGGCAATGAACTTCCTCGTCTGGTATACGTGTCTCGTGAAAAACGTCCTGGATTCCAACATCACAAAAAGGCTGGTGCCATGAATGCG
CTTGTTCGTGTATCAGCTGTCCTTACTAATGGGCAATACTTGTTGAACCTTGATTGTGATCACTACATCAACAATAGCAAGGCTCTCCGAGAAGCTATGTGCTTCC
TTATGGACCCAAACCTAGGAAGGAGTGTCTGTTATGTCCAATTCCCTCAGAGGTTCGATGGCATTGATAGGAATGACCGATATGCAAACAGGAACACTGTGTTT
TTCGATATTAACTTGAGAGGTCTTGATGGCCTTCAAGGACCAGTTTATGTGGGAACTGGTTGTGTGTTTAACAGAACTGCCCTATATGGTTATGAGCCTCCAATC
AAAAAGAAAAAGCCAGGCTTCTTCTCTTCACTCTGTGGGGGAAGGAAAAAGACATCAAAATCTAAGAAGAAGAGCTCGGAAAAGAAGTCACACAAACATGCAG
ACAGTTCTGTGCCAGTATTTAATCTTGAAGATATAGAGGAAGGGATTGAAGGTTCTCAATTTGATGATGAGAAATCGCTGATTATGTCTCAAATGAGCTTGGAG
AAGAGATTTGGCCAGTCCAGTGTTTTTGTAGCCTCTACTCTGATGGAATATGGTGGTGTTCCACAATCTGCAACTCCAGAATCTCTTCTGAAAGAAGCTATCCATG
TCATCAGTTGTGGCTATGAGGACAAAACTGACTGGGGATCTGAGATTGGATGGATCTATGGTTCTGTTACAGAAGATATTCTCACTGGGTTCAAGATGCATGCT
CGAGGCTGGCGATCGATCTACTGCATGCCTAAGCGACCAGCTTTCAAGGGATCTGCTCCTATCAACCTTTCAGATCGTCTGAATCAAGTGCTTCGATGGGCTCTT
GGTTCCATTGAAATTCTATTCAGCAGGCATTGTCCCATATGGTATGGCTATGGAGGACGGCTTAAATTCCTGGAGAGATTTGCTTATGTGAATACCACAATTTAT
CCACTCACCTCAATCCCACTCCTCCTATACTGCATATTGCCAGCTGTTTGTCTTCTCACTGGGAAGTTCATCATCCCAGAGATTAGTAACTTTGCGAGTATTTGGTT
TATATTGCTATTTATTTCTATCTTTGCCACTGGTATCCTTGAGATGAGGTGGAGTGGTGTTGGCATTGATGAATGGTGGAGGAATGAACAGTTTTGGGTTATTGG
TGGTATTTCTGCCCATTTATTTGCTGTCTTCCAAGGTCTCCTGAAAGTACTTGCTGGTATTGACACCAGCTTCACTGTCACCTCTAAGGCCACTGATGAAGAAGGC
GATTTTGCCGAGCTCTACATGTTCAAGTGGACAACGCTTCTGATCCCACCAACCACTATTTTGATCATCAACCTGGTTGGTGTGGTTGCCGGCATTTCCTATGCGA
TCAACAGCGGCTACCAGTCGTGGGGGCCTCTCTTTGGGAAACTCTTCTTTGCCTTCTGGGTGATTGTCCACTTGTACCCCTTTCCTCAAGGGTCTCATGGGGAGG
CAGAACCGCACGCCCACCATTGTTGTCGTCTGGGCCATCCTACTCGCATCGATCTTCTCCCTGCTGTGGGTTCGCATCGATCCATTCACCACCCGGGTCACTGGTC
CTG

>SGCesA7
AGAGGATCCACCCACTTCCATATGCGGATCCCAGTTTACCTGTCCAACCAAGGTCCATGGACCCATCCAAGGATCTTGCTGCGTATGGATATGGTAGTGTTGCAT
GGAAGGAGAGGATGGAGAGCTGGAAGCAGAAGCAGGAGAGGATGCACCAGATGAGGAATGATGCCGGTGGTGATGATGGTGGTGATGACGATCTTCCACTA
ATGGATGAAGCAAGACAACCACTGTCCAGGAAAATTCCAATTCCATCAAGCCAGATTAATCCATATAGGATGATTATCATTATTCGGCTTGTGGTTTTGGGGTTC
TTCTTCCACTATCGAGTGATGCATCCAGTGAACGATGCATTTGCTTTGTGGCTCATATCTGTTATCTGTGAAATTTGGTTTGCCATGTCTTGGATCCTTGATCAATT
CCCAAAATGGTTCCCTATTGAGAGAGAGACATACTTAGACCGGCTGTCATTGAGGTTCGACAAGGAAGGCCAGCCATCTCAACTTGCTCCAATCGATTTCTTTGT
CAGTACAGTCGATCCATTAAAGGAACCTCCTTTGGTCACAGCAAATACTGTTCTATCTATCCTTGCGGTGGATTATCCAGTTGATAAGGTTTCTTGCTATGTTTCTG
ATGATGGTGCTGCAATGCTAACATTTGAAGCGTTGTCTGAAACATCCGAATTTGCAAAGAAATGGGTTCCTTTCTGCAAAAGGTTCAATATTGAACCTCGTGCTC
CAGAGTGGTACTTCCAACAGAAAATAGACTACTTGAAAGATAAGGTGGCGGCAAGCTTTGTTGGGGAGAGGAGAGCGATGAAGAGAGAGTATGAGGAATTCA
AGGTCAGAATCAATGCATTGGTTGCTAAAGCCCAGAAAGTTCCTGAAGAAGGATGGACAATGCAAGATGGGACCCCCTGGCCTGGAAACAATGTTCGTGATCA
TCCTGGAATGATTCAGGTCTTCCTTGGCCAAAGTGGAGGTCTTGATTGCGAGGGAAATGAGCTACCACGATTGGTTTATGTTTCAAGAGAAAAACGACCAGGCT
ATAACCATCATAAGAAAGCTGGTGCTATGAACGCATTGGTCAGAGTCTCTGCTGTACTAACAAATGCTCCCTATTTGTTGAACTTGGATTGTGATCACTACATCAA
CAACAGCAAGGCTATAAAGGAAGCAATGTGTTTTATGATGGATCCTTTATTGGGAAAGAAGGTGTGCTATGTGCAGTTCCCTCAAAGATTTGATGGGATTGATC
GTCATGATCGATATGCTAACAGGAATGTCGTCTTTTTCGATATCAATATGAAAGGTTTGGATGGTATTCAAGGCCCAATCTATGTCGGTACTGGATGTGTATTTA
GAAGGCAGGCACTATATGGTTATGATGCCCCTAAATCGAAAAAGCCACCATCAAGGACTTGCAATTGCTGGCCAAAGTGGTGCTTTTGCTGTTGCTGCTGTGGT
AACAGAAAGCACAAGAAGAAGACCACCAAACCTAAAACAGACAAGAAGAAAAAATTATTATTTTTCAAGAAAGAAGAAAATCAATCCCCTGCATATGCTCTTGG
TGAGATTGATGAAGGTGCTCCAGGTGCTGAAAATGAAAAGGCTGGTATTGTAAATCAACAAAAATTAGAAAAGAAATTTGGTCAGTCTTCTGTTTTTGTCACGT
CCACACTTCTTGAGAATGGTGGGACCTTGAAGAGTGCAAGTCCAGCTTCTCTTTTGAAAGAAGCCATACATGTCATCAGTTGTGGTTATGAAGACAAGACTGACT
GGGGAAAAGAGATTGGCTGGATCTATGGATCAGTCACAGAAGATATTCTAACTGGCTTCAAGATGCATTGTCATGGTTGGCGGTCCATTTACTGCATACCTAAA
CGACCTGCATTCAAAGGTTCTGCACCTCTGAATCTTTCAGATCGTCTTCACCAGGTTCTTCGGTGGGCTCTTGGGTCTATTGAAATTTTCTTCAGCAACCATTGCCC
TCTTTGGTATGGGTATGGTGGTGGTTTGAAATTTTTGGAAAGATTTTCCTACATCAACTCCATCGTATATCCTTGGACGTCTATTCCTCTCTTGGCTTATTGTACAT
TGCCTGCCATCTGTTTGCTGACAGGAAAATTTATCACTCCAGAGCTAAACAATGTTGCCAGCCTCTGGTTCATGTCACTTTTTATCTGCATCTTTGCTACAAGCATA

CTAGAAATGAGATGGAGTGGTGTTGGCATTGATAATTGGTGGAGGAATGAGCAGTTTTGGGTCATTGGAGGTGTGTCCTCGCACCTTTTTGCTGTGTTCCAAGG
ACTTCTCAAGGTCATAGCTGGTGTGGATACAAGCTTCACTGTGACATCAAAGGGTGGTGACGATGAAGAGTTCTCAGAGCTATATACATTCAAGTGGACAACCT
TACTAATACCTCCAACCACTCTGCTCTTGTTGAACTTCATTGGTGTGGTTGCTGGTGTTTCAAATGCTATCAACAACGGATATGAATCATGGGGCCCTCTATTCGG
GAAGCTCTTCTTTGCATTCTGGGTGATTGTCCATCTGTATCCGTTCCTGAAAGGTTTGGTTGGAAGGCAAAACAGGACACCAACAATTGTCATTGTCTGGTCCATT
CTGCTGGCTTCAATCTTCTCACTCCTTTGGGTCCCGGATCGATCCTTTCCTAGCAAAAAATGATGGCCCGCTTCTTGAG
