# Supplementary material for: Selection and Validation of Reference Genes for Gene Expression Analysis in Switchgrass (Panicum virgatum) Using Quantitative Real-Time RT-PCR
Source: PLoS One. 2014 Mar 12;9(3):e91474. doi: 10.1371/journal.pone.0091474 (PMC3951385; doi:10.1371/journal.pone.0091474)
Supplement: File S2 — Alignment of the partial-length deduced amino acid sequence of putative P. virgatum cellulose synthase proteins. Amino acid sequence alignment of the highly conserved U1, U2 and U3/4 regions of the predicted SGCesAs. Domains are indicated below the sequence as follows: shaded amino acid with light gray are U domains, the D, D, D, QXXRW motif is indicated in red, the conserved region is underlined with light gray shading within a box, and the variable region (HVR-II) with dark gray shading without a box. (PDF) [file pone.0091474.s002.pdf]

```
SGCesA3         LWLISVICEIWFAMSWILDQFPKWFPIERETYLDRLTLRFDKEGQPSQLAPVDFFVSTVD 60
SGCesA7         LWLISVICEIWFAMSWILDQFPKWFPIERETYLDRLSLRFDKEGQPSQLAPIDFFVSTVD 60
SGCesA2         LWLISVICEIWFAMSWILDQFPKWLPIERETYLDRLSLRFDKEGRPSQLAPVDFFVSTVD 60
SGCesA5         LWLLSVICEIWFALSWILDQFPKWFPINRETYLDRLALRYDREGEPSQLAAVDIFVSTVD 60
SGCesA6         ------------------------------------------------------------
SGCesA4         LWLVSVICEVWFALSWLLDQFPKWHPINRETYLDRLALRYDREGEPSQLAPIDVFVSTVD 60
SGCesA1         LWMTSVICEIWFGFSWILDQFPKWYPINRETYVDRLIARYG-DGEDSGLAPVDFFVSTVD 59

SGCesA3         PMKEPPLVTANTVLSILAVDYPVDKVSCYVSDDGAAMLTFEALSETSEFAKKWVPFCKKY 120
SGCesA7         PLKEPPLVTANTVLSILAVDYPVDKVSCYVSDDGAAMLTFEALSETSEFAKKWVPFCKRF 120
SGCesA2         PSKEPPLVTANTVLSILAVDYPVEKVSCYVSDDGAAMLTFEALSETSEFAKKWVPFCKKF 120
SGCesA5         PMKEPPLVTANTVLSILAVDYPVDKVSCYVSDDGAAMLTFDALAETSEFARKWVPFVKKY 120
SGCesA6         ------------------------------------------------------------
SGCesA4         PLKEPPLITANTVLSILAVDYPVDKVSCYVSDDGSAMLTFEALSETAEFARKWVPFCKKH 120
SGCesA1         PLKEPPLITANTVLSILAVDYPVEKISCYVSDDGSAMLTFESLAETAEFARKWVPFCKKY 119
                                                  --------------------------

SGCesA3         SIEPRAPEWYFQQKIDYLRDKVATNFVRERRAMKREYEEFKVRINALVAKAQKVPEEGWT 180
SGCesA7         NIEPRAPEWYFQQKIDYLKDKVAASFVGERRAMKREYEEFKVRINALVAKAQKVPEEGWT 180
SGCesA2         NIEPRAPEWYFQQKIDYLKDKVAASFVRDRRAMKREYEEFKVRINALVAKAQKVPEEGWT 180
SGCesA5         NIEPRAPEWYFSQKIDYLKDKVHPSFVKDRRAMKREYEEFKIRINGLVAKAQKVPEEGWI 180
SGCesA6         ----------------------------------------------LVAKAQKVPEEGWI 14
SGCesA4         NIEPRAPEFYFAQKIDYLKDKIQPSFVKERRAMKREYEEFKVRINALVAKAQKVPEEGWT 180
SGCesA1         AIEPRAPEFYFSQKIDYLKDKIHPSFVKERRAMKRDYEEYKVRINALVAKAQKTPDEGWI 179
                -----------------------------------------------*******.*:***

SGCesA3         MQDGTPWPGNNVRDHPGMIQVFLGQSGGHDVEGNELPRLVYVSREKRPGYNHHKKAGAMN 240
SGCesA7         MQDGTPWPGNNVRDHPGMIQVFLGQSGGLDCEGNELPRLVYVSREKRPGYNHHKKAGAMN 240
SGCesA2         MQDGSPWPGNNVRDHPGMIQVFLGQSGGHDVEGNELPRLVYVSREKRPGYNHHKKAGAMN 240
SGCesA5         MQDGTPWPGNNTRDHPGMIQVFLGHSGGLDTEGNELPRLVYVSREKRPGFQHHKKAGAMN 240
SGCesA6         MQDGTPWPGNNTRDHPGMIQVFLGHSGGLDAEGNELPRLVYVSREKRPGFQHHKKAGAMN 74
SGCesA4         MADGTAWPGNNTRDHPGMIQVFLGHSGGLDTDGNELPRLVYVSREKRPGFQHHKKAGAMN 240
SGCesA1         MQDGTPWPGNNPRDHPGMIQVFLGETGARDFDGNELPRLVYVSREKRPGYQHHKKAGAMN 239
                * **:.***** ************.:*. * :*****************::*********

SGCesA3         ALVRVSAVLTNAPYMLNLDCDHYINNSKAIKEAMCFMMDPLLGKKVCYVQFPQRFDGIDR 300
SGCesA7         ALVRVSAVLTNAPYLLNLDCDHYINNSKAIKEAMCFMMDPLLGKKVCYVQFPQRFDGIDR 300
SGCesA2         ALVRVSAVLSNAPYLLNLDCDHYINNSKAIKEAMCFMMDPLVGKKVCYVQFPQRFDGIDR 300
SGCesA5         ALVRVSAVLTNGQYMLNLDCDHYINNSKALREAMCFLMDPNLGRSVCYVQFPQRFDGIDR 300
SGCesA6         ALVRVSAVLTNGQYLLNLDCDHYINNSKALREAMCFLMDPNLGRSVCYVQFPQRFDGIDR 134
SGCesA4         ALIRVSAVLTNGAYLLNVDCDHYFNSSKALREAMCFMMDPALGRKTCYVQFPQRFDGIDL 300
SGCesA1         ALVRVSAVLTNAPYILNLDCDHYVNNSKAVREAMCFMMDPTVGRDVCYVQFPQRFDGIDR 299
                **:******:*. *:**:*****.*.***::*****:*** :*:..*************

SGCesA3         HDRYANRNVVFFDINMKGLDGIQGPIYVGTGCVFRRQALYGYDAPKTKKPPSRTCNCWPK 360
SGCesA7         HDRYANRNVVFFDINMKGLDGIQGPIYVGTGCVFRRQALYGYDAPKSKKPPSRTCNCWPK 360
SGCesA2         HDRYANRNVVFFDINMKGLDGIQGPIYVGTGCVFRRQALYGYDAPKTKKPPSRTCNCWPK 360
SGCesA5         NDRYANRNTVFFDINLRGLDGIQGPVYVGTGCVFNRTALYGYEPPIKQ-KKKGGF----- 354
SGCesA6         NDRYANRNTVFFDINLRGLDGLQGPVYVGTGCVFNRTALYGYEPPIK--KKKPGF----- 187
SGCesA4         HDRYANRNIVFFDINMKGLDGIQGPVYVGTGCCFNRQALYGYDPVLTEADLEPNI----- 355
SGCesA1         SDRYANRNVVFFDVNMKGLDGIQGPVYVGTGCCFYRQALYGYGPPSLPALPKSSICS--- 356
                 ******* ****:*::****:***:****** * * *****.       .---------

SGCesA3         W-ICCCCFGNRKTKKKTKRSKPKFEKIKKLFKKKENQAPAYALGEIDEAAPG--AENEKA 417
SGCesA7         WCFCCCCCGNRKHKKKTTKPKTDKKKKLLFFKKEENQSPAYALGEIDEGAPG--AENEKA 418
SGCesA2         WCLSCFCCRN-KSKKKTTKPKTEKKKKRLFFKKAENPSPAYALGEIEEGAPG--ADVEKA 417
SGCesA5         --LSSLCGGRKKASKSKKGSDKKKSQ-----KHVDSSVPVFNLEDIEEGVEGAGFDDEKS 407
SGCesA6         --FSSLCGGRKKTSKSKKKSSEKKSH-----KHADSSVPVFNLEDIEEGIEGSQFDDEKS 240
SGCesA4         --VVKSCCGRRKKKNKSYMDSQSRIM-----KRTESSAPIFNMEDIEEGIEG--YEDERS 406
SGCesA1         --WCCCCCPKKKAERSEREINRDSRR-------EDLESAIFNLREIDNYDEY-----ERS 402
                ------*  . * ...    . .           :   . : : :*::         *::

SGCesA3         SIVNQQKLEKKFGQSSVFVASTLLENGGTLKSASPASLLKEAIHVISCGYEDKTDWGKDI 477
SGCesA7         GIVNQQKLEKKFGQSSVFVTSTLLENGGTLKSASPASLLKEAIHVISCGYEDKTDWGKEI 478
SGCesA2         GIVNQQKLEKKFGQSSVFVASTLLENGGTLKSASPASLLKEAIHVISCGYEDKTDWGKEI 477
SGCesA5         LLMSQMSLEKRFGQSAAFVASTLMEYGGVPQSATPESLLKEAIHVISCGYEDKTEWGTEI 467
SGCesA6         LIMSQMSLEKRFGQSSVFVASTLMEYGGVPQSATPESLLKEAIHVISCGYEDKTDWGSEI 300
SGCesA4         VLMSQRKLEKRFGQSPIFIASTFMTQGGIPPSTNPASLLKEAIHVISCGYEDKTEWGKEI 466
SGCesA1         MLISQMSFEKTFGLSSVFIESTLMENGGVPESANPSTLIKEAIHVISCGYEEKSEWGKEI 462
```

```
                    -::.* .:** ** *. *: **::  **   *:.* :*:************:*::**.:*

SGCesA3             GWIYGSVTEDILTGFKMHCHGWRSIYCIPKWAAFKGSAPLNLSDRLHQVLRWALGSIEIF 537
SGCesA7             GWIYGSVTEDILTGFKMHCHGWRSIYCIPKRPAFKGSAPLNLSDRLHQVLRWALGSIEIF 538
SGCesA2             GWIYGSITEDILTGFKMHCHGWRSIYCIPKRPAFKGSAPLNLSDRLHQVLRWALGSVEIF 537
SGCesA5             GWIYGSVTEDILTGFKMHARGWRSIYCMPKRPAFKGSAPINLSDRLNQVLRWALGSVEIL 527
SGCesA6             GWIYGSVTEDILTGFKMHARGWRSIYCMPKRPAFKGSAPINLSDRLNQVLRWALGSIEIL 360
SGCesA4             GWIYGSVTEDILTGFKMHARGWQSIYCMPPRPCFKGSAPINLSDRLNQVLRWALGSVEIL 526
SGCesA1             GWIYGSVTEDILTGFKMHCRGWRSIYCMPVRPAFKGSAPINLSDRLHQVLRWALGSVEIF 522
                    ******:***********.:**:****:*  ..******:******:*********:**:

SGCesA3             FSNHCPLWYGYGG-GLKFLERFSYINSIVYPWTSIPLLAYCTLPAICLLTGKFITPELDN 596
SGCesA7             FSNHCPLWYGYGG-GLKFLERFSYINSIVYPWTSIPLLAYCTLPAICLLTGKFITPELNN 597
SGCesA2             FSKHCPLWYGYGG-GLKFLERFSYINSIVYPWTSIPLLAYCTLPAICLLTGKFITPELTN 596
SGCesA5             FSRHCPLWYGYGG-RLKFLERFAYINTTIYPLTSIPLLIYCVLPAICLLTGKFIIPEISN 586
SGCesA6             FSRHCPIWYGYGG-RLKFLERFAYVNTTIYPLTSIPLLLYCILPAVCLLTGKFIIPEISN 419
SGCesA4             LSRHCPIWYGYNG-RLKLLERLAYINTIVYPITSIPLIAYCVLPAICLLTNKFIIPEISN 585
SGCesA1             LSRHCPLWYGYGGGRLKWLQRLSYINTIVYPFTSLPLIAYCCLPAICLLTGKFIIPTLSN 582
                    :*.***:****.*  ** *:*::*:*: :** **:**: ** ***:****.*** * : *

SGCesA3             VASLWFMSLFICIFATGILEMRWSG 621
SGCesA7             VASLWFMSLFICIFATSILEMRWSG 622
SGCesA2             AASIWFMALFICIFITGILEMRWSG 621
SGCesA5             FASIWFISLFLSIFATGILEMRWSG 611
SGCesA6             FASIWFILLFISIFATGILEMRWSG 444
SGCesA4             YAGMFFILLFASIFATGILELRWSG 610
SGCesA1             AATIWFLGLFMSIILTSVLELRWSG 607
                     * ::*: ** .*: *.:**:****
```
