# Supplementary material for: Selection and Validation of Reference Genes for Gene Expression Analysis in Switchgrass (Panicum virgatum) Using Quantitative Real-Time RT-PCR
Source: PLoS One. 2014 Mar 12;9(3):e91474. doi: 10.1371/journal.pone.0091474 (PMC3951385; doi:10.1371/journal.pone.0091474)
Supplement: Table S1 — Primer sequence, amplicon and efficiency of the SGCesA genes evaluated in this study. (PDF) [file pone.0091474.s003.pdf]

Gene name	Sequence of forward (F) and reverse (R) primers	Length of the amplified fragment (bp)	Primer efficiency
**SGCesA1**	F 5’-AAGAAGAAGGCTGAAAGAAGTGAGA -3’	100	1.028
	F 5’-CGTAGTTGTCAATTTCCCGAAGA -3’		
**SGCesA2**	F 5’-GCAGTTCCCTCAGAGGTTTGAT-3’	93	1.020
	F 5’-AGTGCCTGCCGTCTAAAGACA-3’		
**SGCesA3**	F 5’-TCTTCAGAAGGCAGGCATTA-3’	100	0.990
	F 5’-CAGCAACAGCAAATCACCACT-3’		
**SGCesA4**	F 5’-CTTTGTACGGCTATGATCCTGTATTG-3’	90	1.017
	F 5’- TGGGAGCCGAAGATTCTGTT-3’		
**SGCesA5**	F 5’-AGAAGGGTGGTTTCTTGTCATCA-3’	95	0.967
	F 5’-CTGTCCACATGCTTCTGTGACTTT-3’		
**SGCesA6**	F 5’- CCAGGCTTCTTCTCTTCACTCTGT-3’	100	0.993
	F 5’-TTGAGAACCTTCAATCCCTTCCT-3’		
**SGCesA7**	F 5’-AATCGAAAAAGCCACCATCA -3’	100	1.079
	F 5’-CCTGGAGCACCTTCATCAAT-3’		
